# Deterministic processes dominate microbial assembly mechanisms in the gut microbiota of cold-water fish between summer and winter

**DOI:** 10.3389/fmicb.2024.1415931

**Published:** 2024-06-12

**Authors:** Liangliang Xu, Peng Xiang, Xiaoshuai Liu, Luying Zhao, Hanxi Chen, Maohua Li, Zhaobin Song

**Affiliations:** ^1^School of Life Sciences, Neijiang Normal University, Neijiang, China; ^2^Key Laboratory of Bio-Resources and Eco-Environment of Ministry of Education, College of Life Sciences, Sichuan University, Chengdu, China; ^3^Chengdu Academy of Environmental Sciences, Chengdu, China; ^4^Yalong River Hydropower Development Company, Ltd., Chengdu, China; ^5^Hubei Key Laboratory of Three Gorges Project for Conservation of Fishes Chinese Sturgeon Research Institute, China Three Gorges Corporation, Yichang, Hubei, China; ^6^Yangtze Eco-Environment Engineering Research Center, China Three Gorges Corporation, Wuhan, Hubei, China

**Keywords:** cold-water fish, seasonal variation, microbial assembly mechanism, gut bacteria, environment

## Abstract

Exploring the effects of seasonal variation on the gut microbiota of cold-water fish plays an important role in understanding the relationship between seasonal variation and cold-water fish. Gut samples of cold-water fish and environmental samples were collected during summer and winter from the lower reaches of the Yalong River. The results of the 16S rRNA sequencing showed that significant differences were identified in the composition and diversity of gut bacteria of cold-water fish. Co-occurrence network complexity of the gut bacteria of cold-water fish was higher in summer compared to winter (Sum: nodes: 256; edges: 20,450; Win: nodes: 580; edges: 16,725). Furthermore, from summer to winter, the contribution of sediment bacteria (Sum: 5.3%; Win: 23.7%) decreased in the gut bacteria of cold-water fish, while the contribution of water bacteria (Sum: 0%; Win: 27.7%) increased. The normalized stochastic ratio (NST) and infer community assembly mechanisms by phylogenetic bin-based null model analysis (iCAMP) showed that deterministic processes played a more important role than stochastic processes in the microbial assembly mechanism of gut bacteria of cold-water fish. From summer to winter, the contribution of deterministic processes to gut bacteria community assembly mechanisms decreased, while the contribution of stochastic processes increased. Overall, these results demonstrated that seasonal variation influenced the gut bacteria of cold-water fish and served as a potential reference for future research to understand the adaptation of fish to varying environments.

## Introduction

Disentangling the community assembly mechanisms is crucial for understanding the animal adaptation process (Yan et al., 2016; Ning et al., 2019; Xu et al., 2023). Neutral and niche-based theories are important mechanisms to explore the host's microbial assembly mechanisms (Sloan et al., 2006). Neutral theories assume that all individuals are ecologically equivalent and stochastic processes that largely control species dynamics and patterns, including speciation/extinction, migration, and random birth/death (Gravel et al., 2006; Chase and Myers, 2011). On the contrary, niche-based theories postulate that deterministic processes, such as abiotic factors (e.g., pH, temperature) and biotic factors (e.g., predation and competition), can largely control species distribution and persistence (Gravel et al., 2006; Stegen et al., 2013; Ning et al., 2019). An increasing number of models have been developed to determine the relative importance of stochastic and deterministic processes in microbial community assembly based on the neutral and null modeling methods (Stegen et al., 2015; Ning et al., 2019). These models include infer community assembly mechanisms by phylogenetic bin-based null model analysis (iCAMP), beta nearest-taxon index (βNTI) (Stegen et al., 2012), Raup-Crick index (RC_bray_) (Stegen et al., 2013), and normalized stochastic ratio (NST) (Ning et al., 2019). From day 0 to day 30, the gut bacterial community assembly mechanisms of *Schizothorax wangchiachii* significantly increased in the contribution of deterministic processes, while decreasing in the contribution of stochastic processes (Kruskal–Wallis *H*-test; *P* < 0.05) (Xu et al., 2023). However, few studies have comprehensively investigated the ecological processes that regulate the gut microbial community assembly of cold-water fish between different seasons.

The fish gut microbiome plays an important role in helping the host to adapt to seasonal variation (Dehler et al., 2017; Wang et al., 2018; Dulski et al., 2020). Seasonal variation may include a range of water temperature, food availability, pH, and host habitat. Previous studies have reported that seasonal variation influenced the gut's total bacterial abundance and the dominant species of fish (Al-Harbi and Naim Uddin, 2004; Hagi et al., 2004). Differences were identified in the gut bacterial composition of *Salmo salar* between different water temperatures (Neuman et al., 2016). The increase in temperatures (to 21°C) was associated with an increase in *Vibro* spp. and the disappearance of lactic acid bacteria (LAB) and *Acinetobacteri* spp. (Neuman et al., 2016). These results may help fish to adapt to varying environmental temperatures. The relative abundance of Proteobacteria in the gut microbes of *Tinca tinca* decreased from summer (mean level: 57.61 ± 32.57%) to winter (83.49 ±11.90%) (Dulski et al., 2020). Moreover, the results of preparing three-dimensional plots using principal coordinates analysis (PCoA) showed that significant differences were found in the gut microbial structure of *T. tinca* between summer and autumn (Dulski et al., 2020). The change in the pH of water may influence the gut microbial composition of *Leuciscus waleckii*. At the genus level, *Psychrobacter maritimus, Moraxella osloensis*, and *Psychrobacter faecalis* were identified to be the dominant bacteria in the gut of *L. waleckii* inhabiting in high pH environments (pH: 9.4–9.6), while *Aeromonas* and *Ralstonia* were significantly enriched in low pH environments (pH: 7.3–7.9) (Luo et al., 2022). Therefore, exploring the effect of seasonal variation on the cold-water fish gut microbiome could play a key role in understanding the host adaptation processes.

*Schizothorax wangchiachii, Schizothorax kozlovi*, and *Percocypris pingi* are vital economical fish species distributed in the upper reaches of the Yangtze River and its tributaries (Yue, 2000). In this study, the NST and iCAMP models were used to determine the relative importance of stochastic and deterministic processes on the gut bacteria of cold-water fish between summer and winter. The gut samples of cold-water fish with different feeding habits (Herbivorous: SW, *S. wangchiachii*; omnivorous: SK, *S. kozlovi*; carnivorous: PP, *P. pingi*) and environmental samples (water and sediment samples) were collected from the lower Yalong River. The present study aimed to hypothesize that, from summer to winter, the relative importance of deterministic processes on the gut bacterial community will decrease, while the relative importance of stochastic processes will increase.

## Materials and methods

### Sample collection

In total, 30 gut samples and 20 environmental samples (water samples: 10; sediment samples: 10) were collected from summer (sum) and winter (win) in the lower reaches of the Yalong River, Sichuan Province, China (101°64′86.16^′′^; 28°34′14.82^′′^). Each cold-water fish was captured by drift nets in the sampling water area and euthanized with MS-222 (06–1.0 g/L; a chemical used to anesthetize fish samples) to collect the gut content. Water environmental variables were measured in the sampling water areas during both summer and winter. The pH and temperature (TEM) of the water samples were measured using a multiparameter instrument (HI-98130 pH/EC/TDS/°C, HANNA Instruments, Woonsocket, RI, USA), while electrical conductivity (EC) was measured using a conductivity meter (sensION+ MM150 portable meter, Hach). Dissolved oxygen (DO) was determined using a dissolved oxygen analyzer (WTW Multi 3420 Set G, Xylem Inc., Germany), while total dissolved solids (TDS) and salt (SALT) were measured using a multiparameter instrument (HI-98130 pH/EC/TDS/°C, HANNA Instruments, Woonsocket, RI, USA).

For environmental sampling, each sediment sample (3 cm deep and 2.5 cm wide) was collected three times using an aseptic shovel from one sampling site (Chang et al., 2017). Each water sample was collected in three 10-L sterile polyethylene terephthalate (PET) bottles and immediately stored at −20°C. Then, the vacuum pump (pressure: 0.5 MPa; membrane aperture 0.2 μm; and membrane diameter: 10 cm) was used to filter the water sample (Zwart et al., 2002; Liu et al., 2018).

### DNA extraction and 16S rRNA sequencing

The QIAamp DNA Stool Mini Kit (Qiagen, Valencia, CA) was used to extract DNA from gut content, water, and sediment samples according to the manufacturer's instructions. The V4–V5 region of the bacterial 16S rRNA gene was amplified by using universal primers 515F (5′-GTGCCAGCMGCCGCGG-3′) and 907R (5′-CCGTCAATTCMTTTRAGT-3′) (Caporaso et al., 2012). PCR thermocycling conditions were as follows: initial denaturation at 95°C for 5 min, followed by 35 cycles at 95°C for 5 s, annealing at 55°C for 30 s, extension at 72°C for 45 s, and final extension at 71°C for 10 min. All PCR products were purified using a Universal DNA Purification Kit (TIANGEN, China), and the Illumina HiSeq platform (Hiseq2500 PE250) was used for sequencing barcoded V4–V5 amplicons.

### Bioinformatics analysis

Raw pair end reads (PE) were processed using the QIIME 1.9 software package (Caporaso et al., 2010). In quality control, the function Trimmomatic was used to remove low-quality reads (Parameter: ILLUMINACLIP:2:30:10; TRAILING:20, MINLEN:50; SLIDING WINDOW: 50:20) (Bolger et al., 2014), the function search was used for chimerism checks, and the function flash was used for splicing (the minimum length overlap is 10 bp, and the maximum mismatch ratio is 0.2) (Edgar, 2010). After quality control procedures, all clean sequences were clustered into the operational taxonomic units (OTUs) with >97% sequence identity. Each OTU was classified by the annotation against the Silva 132 database (Release 132) (http://www.arb-silva.de; confidence threshold: 0.7) (Quast et al., 2012).

Bar plot and pie chart were generated using the package of *ggplot2* (Wickham, 2011) in R 3.0 to visualize the gut microbial composition of cold-water fish collected during summer and winter at the phylum, family, and genus levels. The Mann–Whitney *U* and Kruskal–Wallis *H*-tests were used to test significant differences in the gut microbial composition and alpha diversity indices of cold-water fish between summer and winter in Stamp software (version 2.1.3) (Parks et al., 2014). Linear discriminant analysis (LDA) effect size (LEfSe) was used to analyze the differences in the gut microbial composition of cold-water fish. The Chao 1 index, observed OTU number index, and Shannon–Wiener index were used to calculate the alpha diversity of the gut microbes of cold-water fish. The box chart was used to visualize the dissimilarity. Bray–Curtis dissimilarity was calculated by the *vegan* package (Dixon, 2003) to assess the beta diversity in the gut and environmental samples between summer and winter. PERMANOVA (number of permutations: 999) based on Bray–Curtis distance was used to analyze the differences in different samples between summer and winter. Nonmetric multidimensional scaling (NMDS) was used to visualize the results (Anderson, 2001). Phylogenetic Investigation of Communities by Reconstruction of Unobserved States (PICRUSt) was used to predict the gut microbial function of cold-water fish between summer and winter. The Mann–Whitney *U*-test and *t*-test (equal variance) were used to calculate significant differences in the KEGG pathways (level1, 2) of the gut microbiota of cold-water fish between summer and winter in Stamp software (version 2.1.3) (Parks et al., 2014).

Redundancy analysis (RDA) was used to examine the relationship between the gut bacteria of cold-water fish and environmental variables in the *vegan* package of R 3.0 (RDA function) (Dixon, 2003). Subsequently, the heatmap was used to identify significant differences between the gut bacteria of cold-water fish (at the phylum, the family, and the genus levels) and environmental variables. To investigate the relationship between gut and environmental microbes (water and sediment microbes), Source-Tracker software (version 0.9.5) was used in this study (Knights et al., 2011). The gut microbes were treated as sinks, while the water and sediment microbes were treated as sources. The Circos graph generated by Circos software was used to show the proportion of the gut, water, and sediment samples. A pie chart was used to visualize the contribution of water and sediment microbes found in the gut microbes of cold-water fish collected between summer and winter.

Co-occurrence networks for the gut bacteria of cold-water fish were constructed based on Spearman rank correlations with OTU abundance (detected in at least 80% of samples). The statistically robust correlations were used in the analysis of co-occurrence networks, and the *P*-value and correlation coefficient were set to 0.001 and 0.6, respectively. To reduce the probability of obtaining false positive results, the Benjamini and Hochberg FDR method was used to adjust all *P*-values in R 3.0 software (Benjamini et al., 2006). Gephi 0.92 software was used to calculate the network diagram parameters and visualize the network (Bastian et al., 2009). Co-occurrence networks were constructed based on Spearman's rank correlation coefficients of the topmost abundant phyla of the gut bacteria of cold-water fish to evaluate the relationship between bacterial community and environmental variables.

### Microbial assembly mechanism analysis

The pNST index (Ning et al., 2019) and iCAMP (Ning et al., 2020) models were used to test the gut bacterial community data of cold-water fish to determine the relative importance of deterministic and stochastic processes on microbial community assembly. In pNST, 0.5 is the boundary point between stochastic processes (>0.5) and deterministic (< 0.5) processes (Ning et al., 2019). The Mann–Whitney *U*-test was used to calculate significant differences in the pNST value of the gut bacteria of cold-water fish between summer and winter. In the iCAMP model, the first step is phylogenetic binning with three binning algorithms based on the distance to abundant taxa, the pairwise distance, and the phylogenetic tree to obtain adequate within-bin phylogenetic signal (Ning et al., 2020). The Mantel test or pNST method was used to compute the relative importance of stochastic processes and estimate the phylogenetic signal of each bin, and then, an optimized phylogenetic signal threshold (ds) and minimal taxa number in a bin (*n*_min_) were chosen (Ning et al., 2020). In the second step, the null model-based phylogenetic metric beta Net Relatedness Index (βNRI) and the taxonomic β-diversity Raup Crick metric (modified RC metric) were used to measure the variations of phylogenetic and taxonomic diversities, respectively (Ning et al., 2020). For each bin, pairwise βNRI < -1.96 or >1.96 indicates homogeneous selection or heterogeneous selection. The value of RC of >0.95 or < -0.95 implied significant deviations from null model expectations (Stegen et al., 2013; Ning et al., 2020). The fraction of pairwise comparisons with |βNRI| ≤ 1.96 and RC > 0.95 indicates the dispersal limitation, while |βNRI| ≤ 1.96 and RC > −0.95 represents homogenizing dispersal. The remaining |βNRI| ≤ 1.96 and |RC| ≤ 0.95 were used to identify the influence of the drift or undominant processes. In the third step, the results of different bins based on an abundance-weighted percentage for each bin or the whole community was incorporated to estimate the relative importance of each ecological process (Ning et al., 2020). Moreover, 1000 randomized permutations and all taxa numbers were used for sbsequent analysis of community assembly structure. The *iCAMP* and *NST* packages in R 3.0 were used to calculate the microbial assembly mechanism analysis (Ning et al., 2019, 2020).

The niche breadth approach (Levins, 1969) was used to quantify the habitat specialization across summer and winter. The niche breadth with larger values indicates that the taxa occupy abundant habitats and are evenly distributed on a large scale, while the lower values indicate that they occupy fewer habitats. The Mann–Whitney *U*-test was used to calculate significant differences in the niche breadth of the gut bacteria of cold-water fish between summer and winter in Stamp software (version 2.1.3) (Parks et al., 2014). Furthermore, the neutral community model (NCM) was used to predict the relationship between OTU detection frequency and their relative abundance across the wider metacommunity (Sloan et al., 2006). In the NCM model, the *R*^2^ indicates the overall fit of the neutral model, and the *N* and *m* represent the metacommunity size and the immigration rate, respectively (Sloan et al., 2006).

## Results

### Gut bacterial community composition and diversity of cold-water fish changed with seasonality

There were significant differences in the gut microbial composition of cold-water fish between summer and winter (Figure 2, Supplementary Figures S1, S2 and Supplementary Table S1). In summer, the dominant phylum was Proteobacteria (relative abundance: 38%), Fusobacteria (38%), and Firmicutes (9%) in the gut microbes of cold-water fish (Figure 2, Supplementary Figure S1 and Supplementary Table S1). However, in winter, Proteobacteria (43%) was dominant in the gut microbiome of cold-water fish, followed by Firmicutes (19%), Fusobacteria (17%), and Cyanobacteria (11%), while the least abundant was Actinobacteria (1%; Figure 2, Supplementary Figure S1 and Supplementary Table S1). The relative abundance of Proteobacteria and Cyanobacteria in the gut microbiota of cold-water fish generally increased (Proteobacteria: sum: 38%; and win: 43%; Cyanobacteria: sum: 7%; win: 11%; Figure 2, Supplementary Figure S1 and Supplementary Table S1). Furthermore, at the species level, the relative abundance of Cyanobacteria in the gut microbiota of cold-water fish was significantly different among SW, SK, and PP (Kruskal–Wallis *H*-test, *P* < 0.05; Supplementary Figure S2). At the genus level, the relative abundance of *Cetobacterium* (sum: 31%; win: 23%), *Clostridium sensu stricto 1* (sum: 5%; win: 2%), and *Rhodobacter* (sum: 4%; win: 3%) in the gut microbiota of cold-water fish decreased from summer to winter, while the *Aeromonas* (sum: 17%; win: 19%) and *Pirellula* (sum: 1%; win: 4%) increased from summer to winter (Supplementary Figure S2 and Supplementary Tables S2, S3).

**Figure 1:**
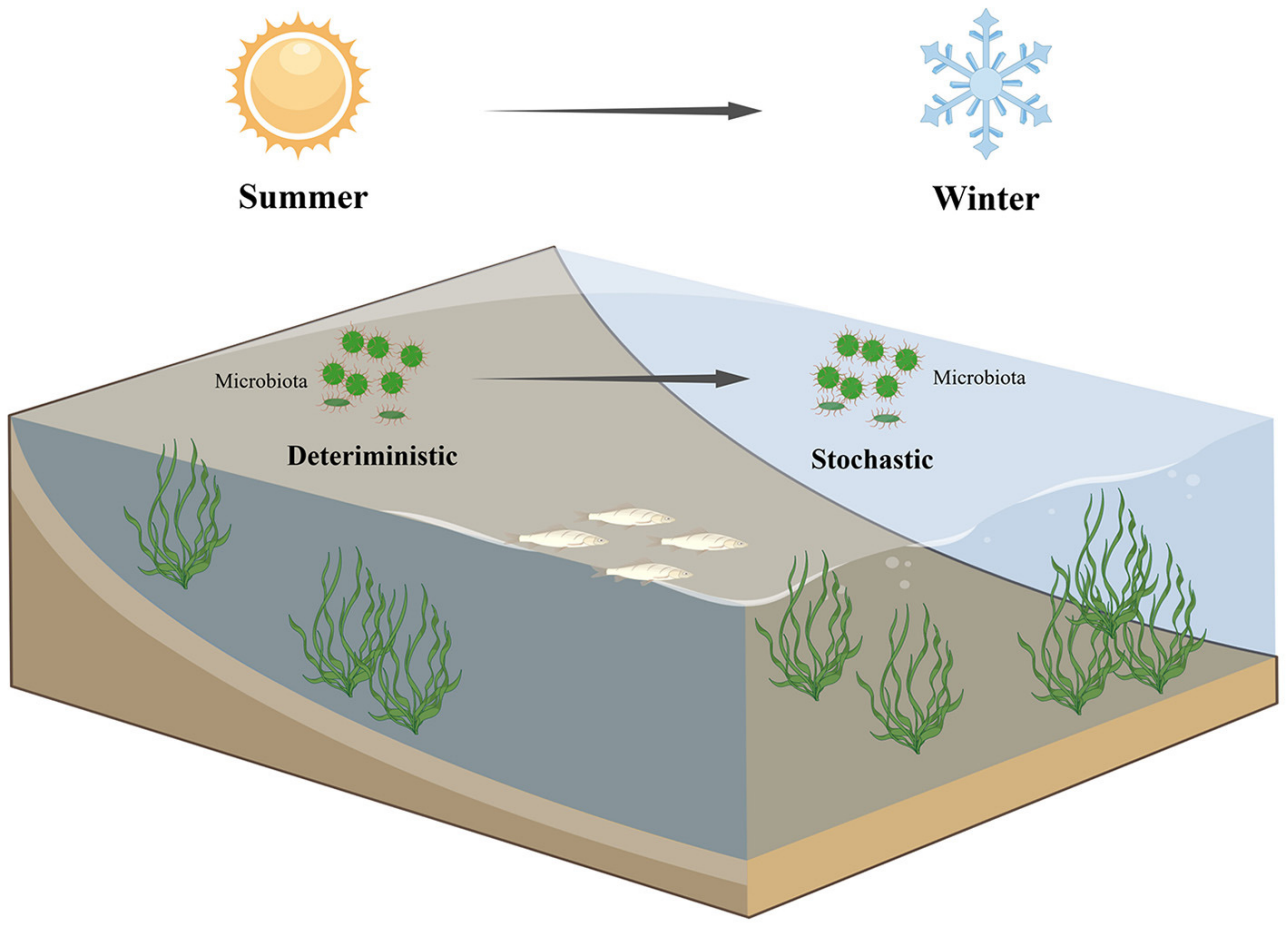
Changes in the gut microbial assembly mechanism of cold-water fish between summer and winter (By Figdraw).

**Figure 2:**
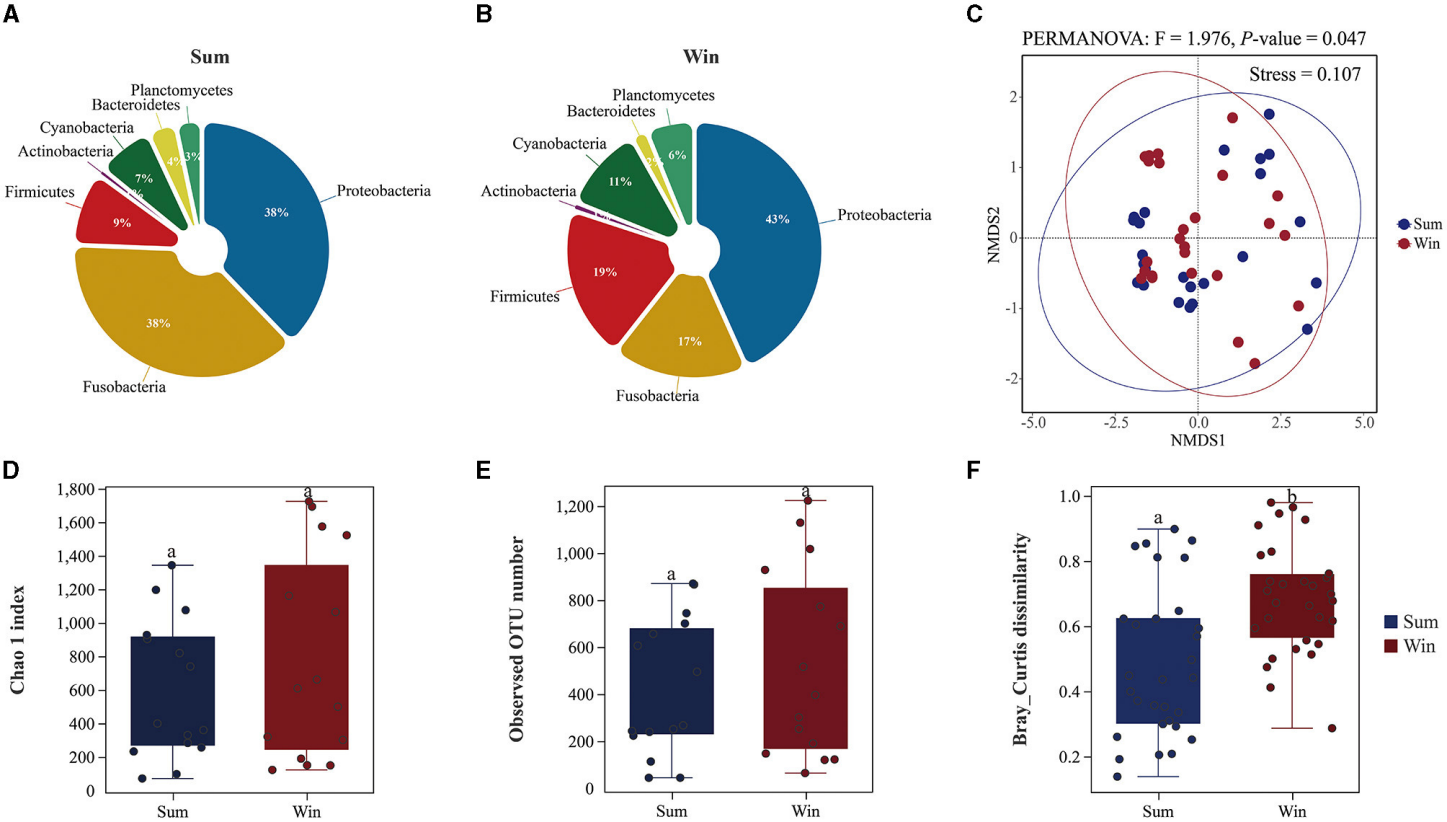
Gut microbial composition and diversity of cold-water fish between summer and winter. **(A, B)** The gut microbial composition of cold-water fish between summer and winter at the phylum level; **(C)** non-metric multidimensional scaling (NMDS) analysis based on Bray–Curtis distances to explore the dissimilarity in the gut bacteria of cold-water fish between summer and winter; **(D, E)** gut microbial alpha diversity of cold-water fish between summer and winter; **(F)** gut microbial beta diversity of cold-water fish between summer and winter.

Significant differences were found in the gut microbial diversity of cold-water fish (PERMANOVA, *P* < 0.05; Figure 1, Supplementary Figure S3 and Supplementary Table S4). The Chao 1 (Mean ± SD: sum: 602 ± 424; win: 782 ± 648) and observed OTU number indices (sum: 424 ± 290; win: 776 ± 382) in the gut microbes of cold-water fish were increased from summer to winter (Figure 2 and Supplementary Table S4). However, in water and sediment microbes, the Chao 1 (water: sum: 3,044 ± 201; win: 1,459 ± 202; sediment: sum: 3,318 ± 378; win: 2,948 ± 189) and observed OTU number indices (water: sum: 2,035 ± 86; win: 2,586 ± 327; sediment: sum: 2,586 ± 327; win: 2,182 ± 181) were significantly decreased from summer to winter (Mann–Whitney *U*-test; both, *p* < 0.01; Supplementary Figure S3 and Supplementary Table S4). At the species level, the Chao 1 and observed OTU number indices in the gut microbes were significantly increased from SW and SK to PP (Kruskal–Wallis *H*-test, both, *p* < 0.05; Supplementary Figure S3).

A non-metric multidimensional scaling plot of the gut bacteria of cold-water fish identified a separation between seasons (summer and winter) and species (SW, SK, and PP; Figure 2C and Supplementary Figure S4). The community structure of the gut microbiota of cold-water fish was significantly different from environmental microbes (water and sediment microbes; ADONIS: *R*^2^ = 0.647; *p* = 0.001; Supplementary Figure S4A). Furthermore, the results of Bray–Curtis dissimilarity in the gut microbiota of cold-water fish significantly increased from summer to winter (sum: 0.68 ± 0.17; win: 0.48 ± 0.23; Mann–Whitney *U*-test, *p* < 0.01; Figure 2). Similarly, the results of Bray–Curtis dissimilarity were significantly different between the three species of the gut microbiota (SW, SK, and PP) and environmental microbes (Supplementary Figure S4B).

### Gut bacterial community assembly of cold-water fish between summer and winter

**Figure 3:**
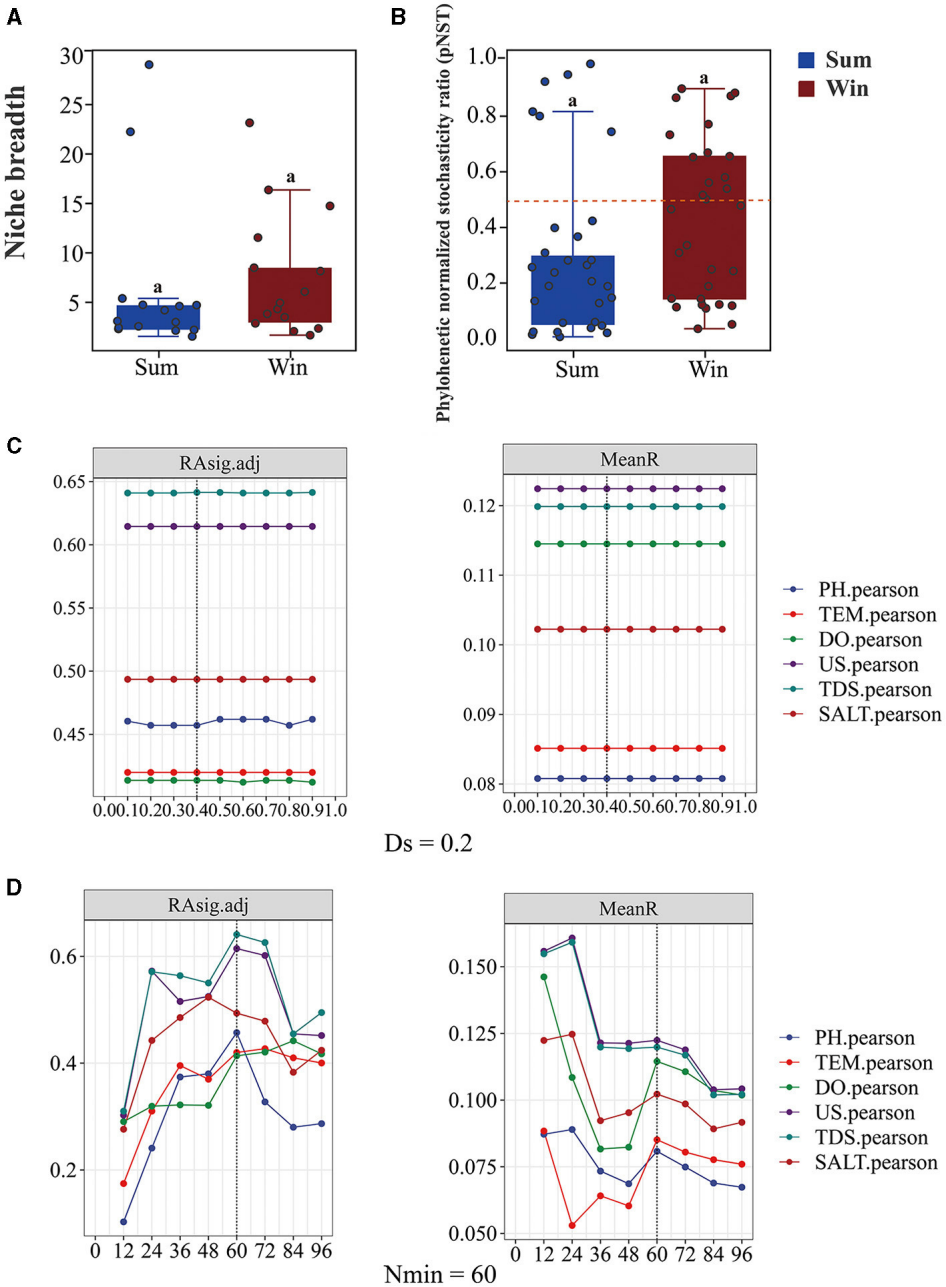
Relative abundance of niche breadth and phylogenetic normalized stochasticity ratio of gut bacteria of cold-water fish between summer and winter. **(A)** Boxplots exhibiting the niche breadth of the gut bacteria of cold-water fish between summer and winter; **(B)** boxplots exhibiting the phylogenetic normalized stochasticity ratio (pNST) of the gut bacteria of cold-water fish between summer and winter; **(C, D)** boxplots of Nmin and DS in the gut bacterial community of cold-water fish. The ST_Dab_ value at different levels of the Nmin (minimal bin size); with Ds equal to 0.2 and the Ds (phylogenetic signal threshold) with Nmin equal to 60, respectively. The Mann–Whitney *U*-test was used to analyze the significant differences in niche breadth and pNST among different samples.

Overall, the value of pNST was below the 50% boundary for gut bacteria of cold-water fish between summer and winter (the value of pNST: sum: 0.3 ± 0.3; win: 0.4 ± 0.2), implying that deterministic processes played a more important role than stochastic processes (Figures 1, 3B and Table 1). At the species level, differences in the value of pNST were found in gut bacteria of SW, SK, and PP between summer and winter (Table 1). For example, in SW and SK, the value of pNST of gut bacteria increased from summer to winter (SW: sum: 0.3 ± 0.1; win: 0.7 ± 0.1; SK: sum: 0.1 ± 0.1; win: 0.3 ± 0.2;), while it decreased in PP (PP: sum: 0.6 ± 0.4; win: 0.3 ± 0.3; Table 1).

**Table 1:** Microbial assembly mechanism of the gut bacteria of cold-water fish and environmental bacteria between summer and winter.

**Group**	**Season**	**Assembly mechanism**	
		**NST**	**Niche breadth**
SW	Sum	0.3 ± 0.1	4.1 ± 1.2
	Win	0.7 ± 0.1	13.2 ± 7.4
SK	Sum	0.1 ± 0.1	2.9 ±1
	Win	0.3 ± 0.2	3.5 ± 1.1
PP	Sum	0.6 ± 0.4	12 ± 13
	Win	0.3 ± 0.3	6 ± 4
Water	Sum	0.7 ± 0.2	37 ± 6
	Win	0.3 ± 0.1	31 ± 4
Sediment	Sum	0.4 ± 0.1	189 ± 89
	Win	0.4 ± 0.1	107 ± 84
All level	Sum	0.3 ± 0.3	6.3 ± 5.0
	Win	0.4 ± 0.2	7.6 ± 5.0
	Sum_Env	0.6 ± 0.2	113 ± 107
	Win_Env	0.4 ± 0.1	69 ± 54

**Figure 4:**
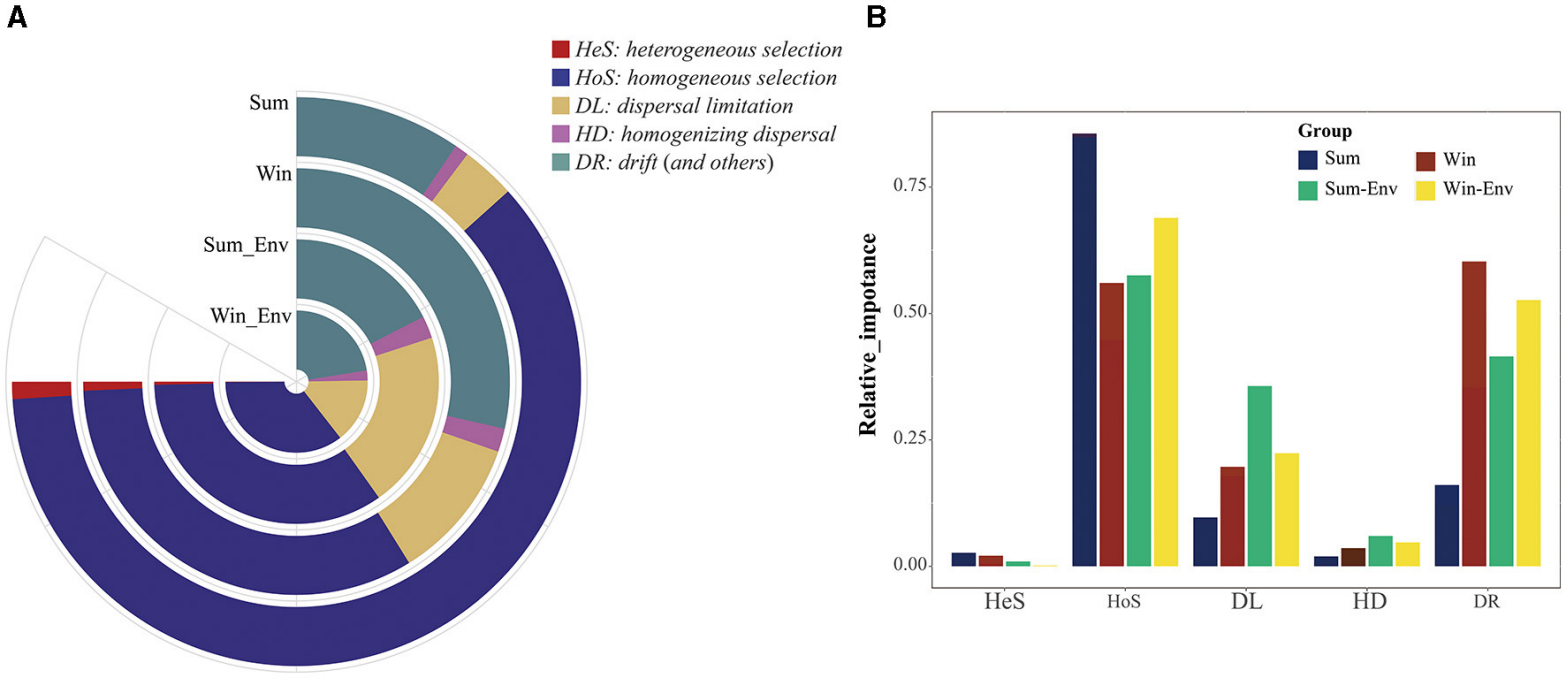
The relative importance of different bacterial community assembly mechanisms of the gut bacteria of cold-water fish and environmental bacteria between summer and winter. **(A)** The area of the ring map represents the proportion of different ecological processes; **(B)** differences of five ecological processes in the bacterial community assembly mechanisms of the gut bacteria of cold-water fish and environmental bacteria between summer and winter.

In the iCAMP model, the most suitable number of Ds and Nmin was 0.2 and 60, respectively (Figures 3C, D). From summer to winter, the results of iCAMP showed that the gut bacterial community assembly mechanisms decreased in the contribution of deterministic processes but increased in the contribution of stochastic processes (Figure 4). Furthermore, the homogeneous selection was the dominant microbial assembly mechanism, followed by drift and dispersal limitation (Figure 4). Similarly, the deterministic processes played a more important role than the stochastic processes in environmental bacteria (Sum_Env and Win_Env; Figure 4). Significant differences were identified in the homogeneous selection mechanism of the gut bacteria of cold-water fish between summer and winter (Mann–Whitney *U*-test, *p* < 0.01; Figure 4). At the species level, homogeneous selection was the dominant microbial assembly mechanism, followed by drift, dispersal limitation, and heterogeneous dispersal, with heterogeneous selection exhibiting the lowest microbial assembly mechanism (Supplementary Figures S5, S6).

The niche breadth and the NCM model were used to estimate the community-level habitat of cold-water fish (Figure 3A and Supplementary Figure S7). The niche breadth of the gut bacteria of cold-water fish increased from summer to winter (sum: 6.3 ± 5.0; win: 7.6 ± 5.0; Figure 3A and Table 1). At the species level, in SW and SK (SW: sum: 4.1 ± 1.2; win: 13.2 ± 7.4; SK: sum: 2.9 ±1; win: 3.5 ± 1.1), the niche breadth increased from summer to winter but decreased in PP (PP: sum: 12 ± 13; win: 6 ± 4; Table 1). Furthermore, the findings of the NCM model showed that a higher *Nm* value of the gut bacteria of cold-water fish was found in winter than in summer (sum: 779; win: 863; Supplementary Figures S7A, B).

### Relationship between the gut bacteria of cold-water fish and environmental variables

**Figure 5:**
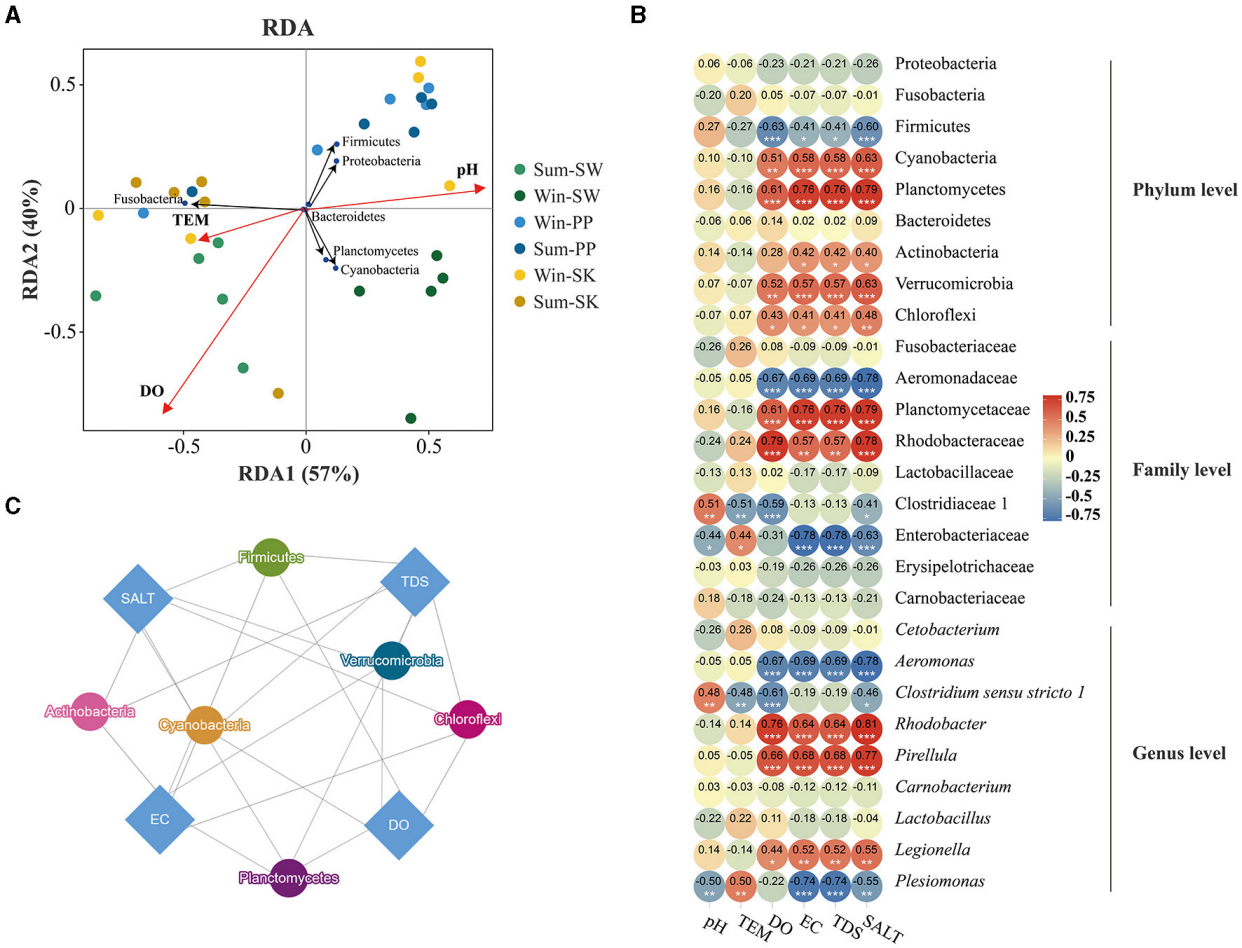
Relationship between environmental variables and gut bacteria of cold-water fish. **(A)** Redundancy analysis (RDA) correlation of skin and gut bacteria of amphibians. Arrows indicated the director and magnitude of each microbial community at the phylum level. The black circle represents phyla. The red circle represents environmental variables. The other circles represent the gut samples of cold-water fish; **(B)** a heatmap illustrating the relationship between environmental variables and the gut bacteria at the phylum, family, and genus levels. The Mann–Whitney *U*-test was employed to assess significant differences in the environmental variables and the gut bacteria of cold-water fish. *P* < 0.001, marked “***”, *P* < 0.01, marked “**”, *P* < 0.05, marked “*”. **(C)** Network of environmental variables and gut bacteria of cold-water fish between summer and winter. The blue diamond represents environmental variables; and the other circles represent phyla. The lines indicate that there is a relationship between bacteria and environmental variables.

**Figure 6:**
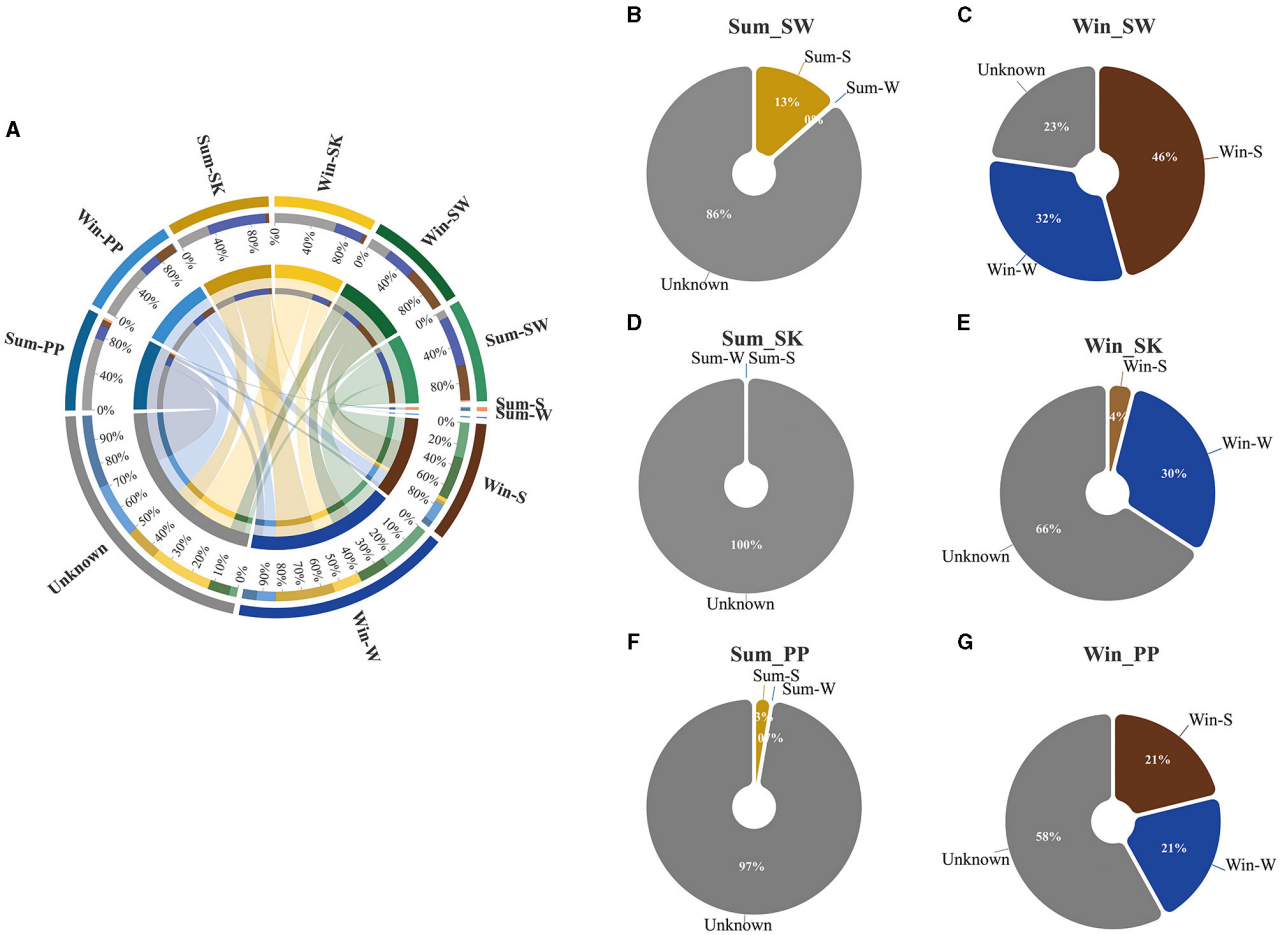
Relationship between the gut bacteria of cold-water fish and environmental bacteria between summer and winter. **(A)** The circle plot represents the contribution of water and sediment bacteria for the gut bacteria of cold-water fish between summer and winter; **(B–G)** Pie charts represent the relative percentage of water, sediment, and unknown for the gut bacteria of cold-water fish between summer and winter.

Differences in the effects of environmental variables on the gut bacteria of cold-water fish (SW, SK, and PP) were identified between summer and winter (Figure 5). In the RDA analysis, the first two RDA axes explained 97% total variance, with RDA1 accounting for 57% of the total variance and RDA2 accounting for 40% of the variance (Figure 5A). Water temperature (TEM), pH, and DO were major environmental variables 322 influencing the gut bacterial community of cold-water fish (Figure 5A). Furthermore, the results of RDA showed that Fusobacteria was positively correlated with TEM, while Firmicutes and Proteobacteria were positively correlated with pH (Figure 5A). The results of the heatmap confirmed these findings (Figure 5B). At the family level, Fusobacteriaceae and Aeromonadaceae were positively correlated with TEM but negatively correlated with pH, DO, EC, TDS, and SALT (Figure 5B). At the genus level, *Clostridium sensu stricto 1* was significantly positively correlated with pH (*p* < 0.05) but significantly negatively correlated with DO (*p* < 0.05; Figure 5B). The results of the network indicated differences in the effects of environmental variables on cold-water fish (Figure 6C). At the phylum level, Firmicutes, Cyanobacteria, Planctomycetes, Verrucomicrobia, Actinobacteria, and Chloroflexi were influenced by the SALT, TDS, EC, and DO (Figure 5C).

The contribution of water and sediment bacteria to gut bacteria of cold-water fish varied between summer and winter (Figure 6). Overall, the contribution of sediment bacteria decreased in the gut bacteria of cold-water fish, while the contribution of water bacteria increased from summer to winter (Figure 6A). For example, in summer, the contribution of sediment bacteria (5.3%) to the gut bacteria of cold-water fish was higher than that of water bacteria (0%; Figures 6B–G). However, in winter, the contribution of water bacteria (27.7%) to the gut bacteria of cold-water fish was higher than that of sediment bacteria (23.7%; Figures 6B–G). Moreover, a higher contribution of Win_Env (Win-W and Win-S bacteria) was noted in the gut microbiota of cold-water fish than Sum_Env (Sum-W and Sum-S bacteria; total contribution percentage: Win_Env vs Sum_Env: 51.3% vs 4.3%; Figures 6B–G).

### Co-occurrence analysis of the gut bacteria of cold-water fish between summer and winter

**Figure 7:**
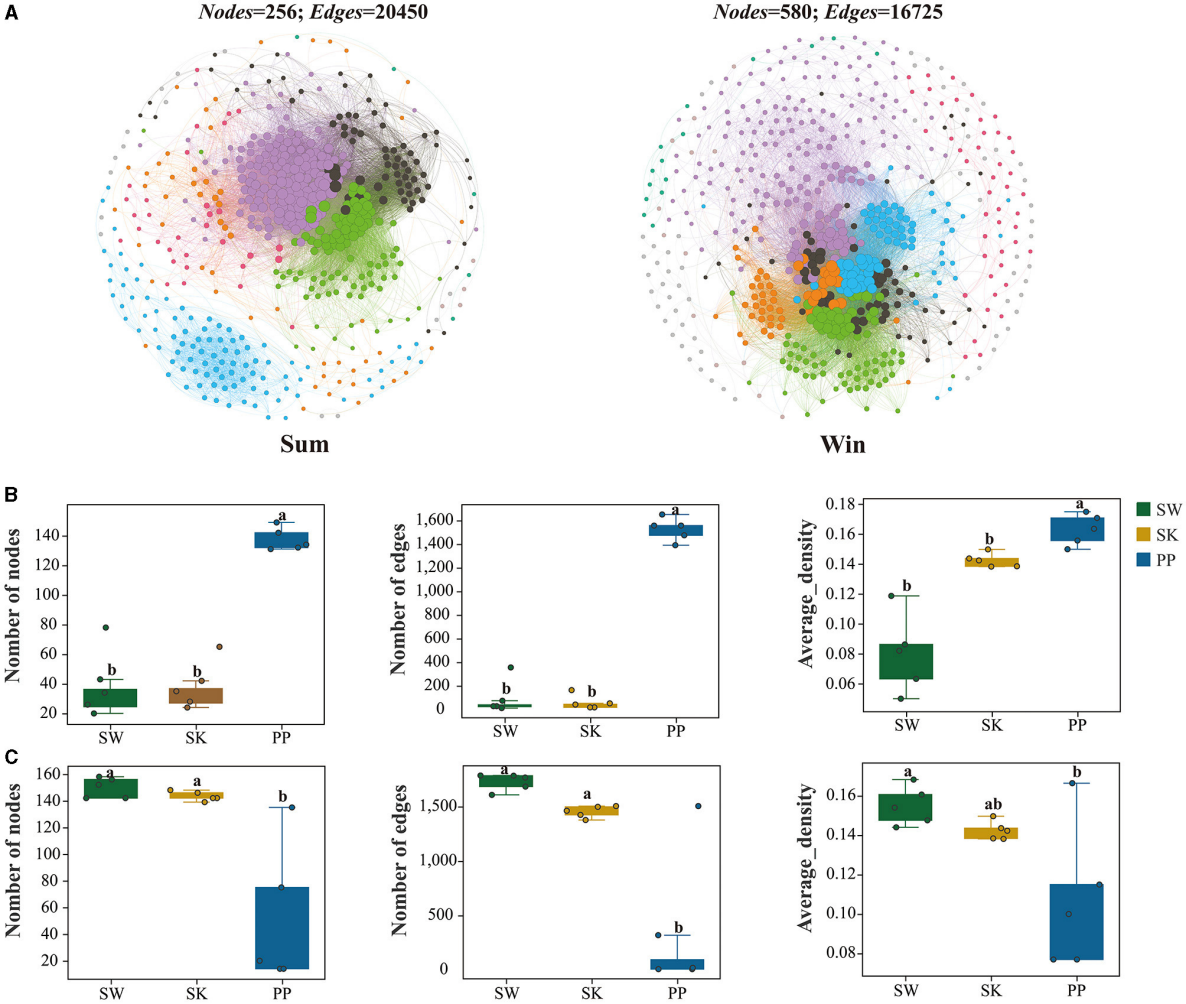
Co-occurrence networks of the gut microbiota of cold-water fish between summer and winter. **(A)** Co-occurrence networks of the gut microbiota of cold-water fish between summer and winter; **(B, C)** variations in the co-occurrence network parameters of the gut microbiota of cold-water fish between summer and winter. The Mann–Whitney *U*-test was employed to assess significant differences in the co-occurrence network parameters.

Overall, the network complexity of the gut bacteria of cold-water fish increased from summer to winter (Figure 7 and Supplementary Figure S9 and Table 2). For example, the number of nodes and edges of the gut bacteria of cold-water fish was higher in summer than in winter (Sum: nodes: 256; edges: 20,450; Win: nodes: 580; edges: 16,725; Figure 7A and Table 2). In the network of environmental bacteria, the network complexity increased from summer to winter (Sum_Env: nodes: 112; edges: 1,119; Win_Env: nodes: 120; edges: 9,769; Figure 7A and Table 2). At the species level, the network complexity in the gut bacteria showed significant differences and increased from SW and SK to PP between summer and winter (Kruskal–Wallis *H*-test; *p* < 0.05; Figure 7B and Supplementary Figure S9). In summer, the highest network complexity was identified in the gut bacteria of PP, followed by the SW, and the lowest was observed in the SK (nodes: SW: 201; SK: 194; PP: 258; edges: SW: 498; SK: 194; PP: 1,857; Figure 7B and Supplementary Figures S9A–C). However, in winter, the highest network complexity was identified in the gut bacteria of SW, followed by the SK, and the lowest was observed in those of the PP (nodes: SW: 750; SK: 717; PP: 688; edges: SW: 8,269; SK: 7266; PP: 7,631; Figure 7B, Supplementary Figures S9D–F).

**Table 2:** Co-occurrence networks parameters of the gut bacteria of cold-water fish and environmental bacteria between summer and winter.

**Co-occurrence networks parameters**	**Sample**			
	**Sum**	**Win**	**Sum_Env**	**Win_Env**
Node	256	580	112	120
Edge	20,450	16,725	1,119	9,769
Positive_edge	556	1,172	1,107	957
Negative_edge	5.6	12	10	20
Average_density	0.10	0.13	0.14	0.12
Transitivity	0.6	0.6	0.63	0.5
Diameter	5.3	5.9	6.1	6.8
Average_path.length	2.1	2.1	2.1	2.4

## Discussion

### Seasonal variation influenced the gut bacterial composition and diversity of cold-water fish

Significant differences were found in the gut bacterial composition of fish in different seasons (Ye et al., 2014; Bazhenov et al., 2019; Dulski et al., 2020; Bereded et al., 2021). Significant differences were identified in the relative abundance of Fusobacteria, Bacteroidetes, and Cyanobacteria of *Oreochromis niloticus* in different months (April, August, July, June, and May). The relative abundance of Fusobacteria was higher in April and August than in other months (Bereded et al., 2021). This result was consistent with our results. Differences in the gut bacterial composition of cold-water fish between summer and winter were observed (Figures 2A, B, and Supplementary Figure S2). For example, the relative abundance of Proteobacteria, Cyanobacteria, and Planctomycetes among the microbes of the gut microbiota of cold-water fish increased from summer to winter (Figures 2A, B). The same result was found at the species level (SW, SK, and PP; Supplementary Figures S1, S2). From summer to winter, the average water temperature of the cold-water fish environment decreased from 15.7 to 7.4°C (Supplementary Table S5). To cope with the decrease of environmental water temperature, cold-water fish may obtain enough nutrients by eating a large amount to adapt to the changes in the environment. Cold-water fish may obtain more food to maintain normal body temperature to cope with the decrease in environmental water temperature. This finding was further supported by the results of PICRUSt (Supplementary Figure S8). A previous study reported that Cyanobacteria is the dominant food for wild *S. wangchiachii* (SW), and it aids in digesting and absorbing nutrients from food (Xu et al., 2022; Zhang, 2022). A higher feeding intensity and fullness index of *S. wangchiachii* were found in winter than in summer (Zhang, 2022). Therefore, an increase in the relative abundance of Cyanobacteria in gut microbes from summer to winter also implies that cold-water fish need to get more food to cope with the challenge of seasonal variation (e.g., water temperature decrease) and for their overwintering and spawning in the next year (Zhang, 2022; Figures 2A, B). Furthermore, through our long-term field survey, we found that more cold-water fish breed from March to June in the following year and concluded that cold-water adult fish need to obtain more food and nutrients in preparation for the upcoming breeding season.

Seasonal variation significantly influenced the gut bacterial diversity of fish (Bazhenov et al., 2019; Dulski et al., 2020; Bereded et al., 2021). A previous study found that significant differences were identified in the alpha diversity indexes of the gut microbes of *O. niloticus* among different seasons. The lower alpha diversity indexes were found in April and August than in other months (Bereded et al., 2021). The alpha diversity of the gut bacteria of cold-water fish increased from summer to winter (Figures 2D, F and Supplementary Figure S3). The beta diversity of the gut bacteria of cold-water fish significantly increased from summer to winter (Figure 2C and Supplementary Figure S4). This phenomenon may be attributed to the different feeding strategies of cold-water fish. A previous study showed that the feeding intensity and the fullness index of *S. wangchiachii* increased from summer to winter (Zhang, 2022). Cold-water fish breeding usually begins in winter and ends in summer, expanding their search range to obtain more food for reproduction. And cold-water fish had more exposure to water bacteria in winter than in summer. The results of gut bacterial composition of cold-water fish confirmed this finding (Figures 2A, B). Overall, seasonal variation significantly influenced the gut bacterial composition and diversity of cold-water fish.

### Seasonal variation influenced the gut bacterial community assembly of cold-water fish

In microbial ecology, studying the gut bacterial community assembly mechanism of fish is essential to understanding the contribution of ecological processes to the structure of microbial communities (Sloan et al., 2006; Stegen et al., 2013; Yan et al., 2016). In this study, the deterministic process dominated the microbial assembly mechanism of cold-water fish between summer and winter (Figures 3, 4). From summer to winter, the microbial assembly analysis showed that gut bacterial community assembly mechanisms decreased in the contribution of deterministic processes (homogeneous selection) but increased in the contribution of stochastic processes (drift; Figure 4). These findings indicated the same environmental selective pressure (e.g., pH, TEM, and DO) for cold-water fish in summer. Similar environmental variables were found in summer (Supplementary Table S5). Moreover, the results of the source-tracker analysis confirmed this finding (Figure 6). However, drift was the dominant factor regulating the gut bacterial assembly mechanism of cold-water fish in winter (Figure 4). This finding may be attributed to the fact that the pressure of the host itself was the main factor influencing the gut bacteria of cold-water fish (Xu et al., 2023). Previous studies have reported that cold-water fish need to get more food to adapt to seasonal variation and overwintering and spawning in the next year (Zhang, 2022). Furthermore, the niche breadth showed an increase in the gut bacteria of cold-water fish from summer to winter (Figure 3A). This result was consistent with the NCM model analysis (Supplementary Figure S7). The gut bacteria of cold-water fish exhibited a greater distribution in winter than in summer. Feeding strategies and biological activity of cold-water fish may explain this finding. In winter, cold-water fish are more likely to acquire food for spawning, and therefore, there are more chances for the gut bacteria to transfer between different environments. However, in summer, cold-water fish have plenty of food, and their breeding period also comes to a halt, and thus, there is less opportunity for gut bacteria to transmit among different environments. Overall, seasonal variation influenced cold-water fish gut bacterial community assembly mechanisms.

### Environmental variables influenced the gut bacteria of cold-water fish between summer and winter

Environment, and season have influenced on the gut bacteria of fish (Dehler et al., 2017; Dulski et al., 2020; Kim et al., 2021), and these findings were consistent with our results. These findings were consistent with our results. Significant differences were identified in the influence of environmental variables on gut bacteria of cold-water fish between summer and winter (Figures 5, 6). It can be noted that the relative abundance of Cyanobacteria in the gut bacteria of cold-water fish had a significantly negative correlation with TEM but a positive correlation with DO, EC, TDS, and SALT (Figure 5). Cyanobacteria is an important food source for cold-water fish (e.g., SW) and may help the host to digest and absorb nutrients (Xu et al., 2022; Zhang, 2022). Thus, water temperature (TEM) was the major factor that influenced the relative abundance of Cyanobacteria in the gut bacteria of cold-water fish between summer and winter.

Cold-water fish prefer fast-flowing environments with plenty of dissolved oxygen (DO) and are good for acquiring food. The result of the source-tracker analysis showed that differences in the contribution of environmental bacteria (water and sediment bacteria) were found between summer and winter (Figure 6). From summer to winter, the contribution of water bacteria to the gut bacteria of cold-water fish increased, while the contribution of sediment bacteria decreased (Figure 6). In summer, cold-water fish are equipped with enough food, and they do not go in search of food. However, cold-water fish need to obtain more food for their overwintering and spawning in the next year in winter (Zhang, 2022). Thus, in winter, cold-water fish may have encountered different environmental factors in water (e.g., DO, TEM, and pH) possibly with different microbes that would contribute to the observed gut bacteria. Overall, environmental variables significantly influenced the gut bacteria of cold-water fish.

### Seasonal variation influenced the gut bacterial network complexity of cold-water fish

Network complexity is an important index to explore the relationship between gut microbes and the environment (Shi et al., 2016). From summer to winter, the network complexity increased in cold-water fish gut bacterial communities (Figure 7 and Supplementary Figure S9). In this study, water temperatures (TEM) decreased in cold-water fish habitats from summer to winter (Supplementary Table S5). This finding implied that cold-water fish faced an extreme challenge (e.g., environmental variation) from summer to winter (Zhang, 2022). Previous studies have shown that an increase in the network complexity of the host's microbial system could enhance its adaptability to the wild environment (Zhao et al., 2021; Xu et al., 2023). Therefore, these findings implied that the higher network complexity may help the gut bacteria of cold-water fish to resist environmental disturbances (i.e., water temperature declines). Moreover, at the species level, the network complexity of the gut bacteria of SW (Herbivorous), SK (Omnivorous), and PP (Carnivorous) increased from summer to winter (Supplementary Figure S9). These findings further support our results. The highest network complexity of gut bacteria was identified in SW. This result illustrated that herbivorous (e.g., SW) gut bacteria required higher network complexity to cope with the challenges posed by seasonal variation (e.g., environmental changes). Overall, these results imply that the greater network complexity of the gut bacteria could play a key role in the adaptation of cold-water fish to seasonal variation.

## Conclusion

This study provides a novel understanding of the relationship between the gut bacteria of cold-water fish and environmental bacteria by exploring the differences between summer and winter. Significant differences occur in the gut bacterial composition, diversity, and network complexity of cold-water fish between summer and winter. Water temperature (TEM), DO, pH were dominant environmental factors that influenced the gut microbiota of cold-water fish. Deterministic processes were the dominant factor regulating the gut bacterial assembly of cold-water fish between summer and winter. From summer to winter, the community assembly mechanisms of the gut bacteria in cold-water fish showed a decrease in the contribution of deterministic processes but an increase in the contribution of stochastic processes. Furthermore, niche breadth was higher in winter than in summer. Together, these results demonstrate that seasonal variation significantly influenced the gut bacterial communities of cold-water fish. In the future, more samples and environmental factors should be collected to systematically explore how cold-water fish adapt to different seasons.

## Data availability statement

The data presented in this study are deposited in the NCBI repository, accession number PRJNA1031347.

## Ethics statement

The animal study was approved by Sichuan Provincial Department of Agriculture and Rural Affairs (2021). The study was conducted in accordance with the local legislation and institutional requirements.

## Author contributions

LX: Writing – review & editing, Writing – original draft, Visualization, Validation, Supervision, Software, Methodology, Investigation, Formal analysis, Data curation, Conceptualization. PX: Writing – review & editing, Software, Investigation. XL: Writing – review & editing, Funding acquisition, Data curation. LZ: Writing – review & editing, Software, Methodology, Investigation, Data curation. HC: Writing – review & editing, Supervision, Software, Methodology, Investigation, Formal analysis. ML: Writing – review & editing, Software, Investigation, Data curation. ZS: Writing – review & editing, Project administration, Funding acquisition, Formal analysis, Data curation.
