# Supplementary material for: Deterministic processes dominate microbial assembly mechanisms in the gut microbiota of cold-water fish between summer and winter
Source: Front Microbiol. 2024 Jun 12;15:1415931. doi: 10.3389/fmicb.2024.1415931 (PMC11216611; doi:10.3389/fmicb.2024.1415931)
Supplement: Supplementary file 1 [file Data_Sheet_1.docx]

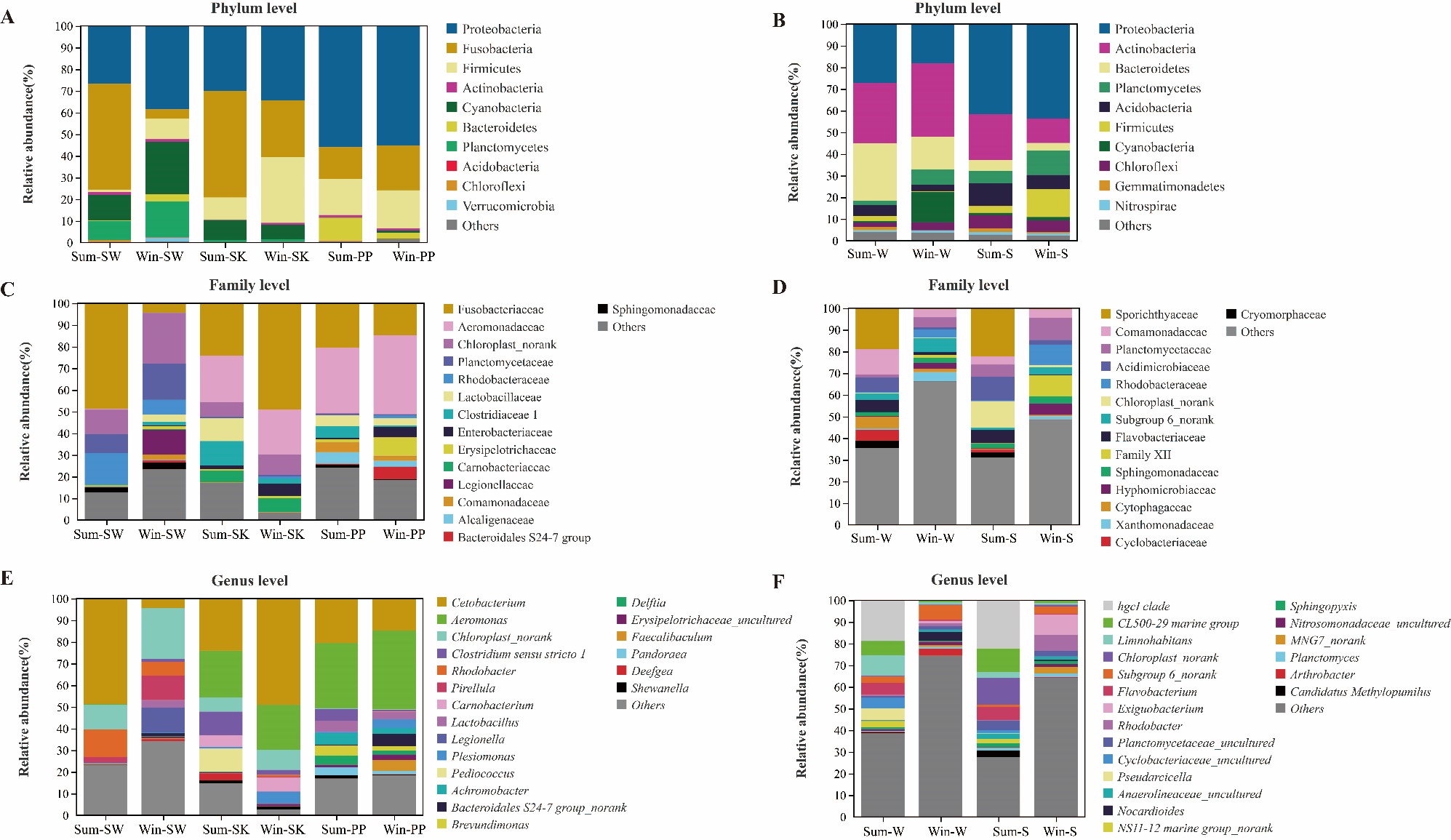


**Figure S1. Gut microbial composition of cold-water fish at phylum (A, B), family (C, D), and genus level (E, F).**


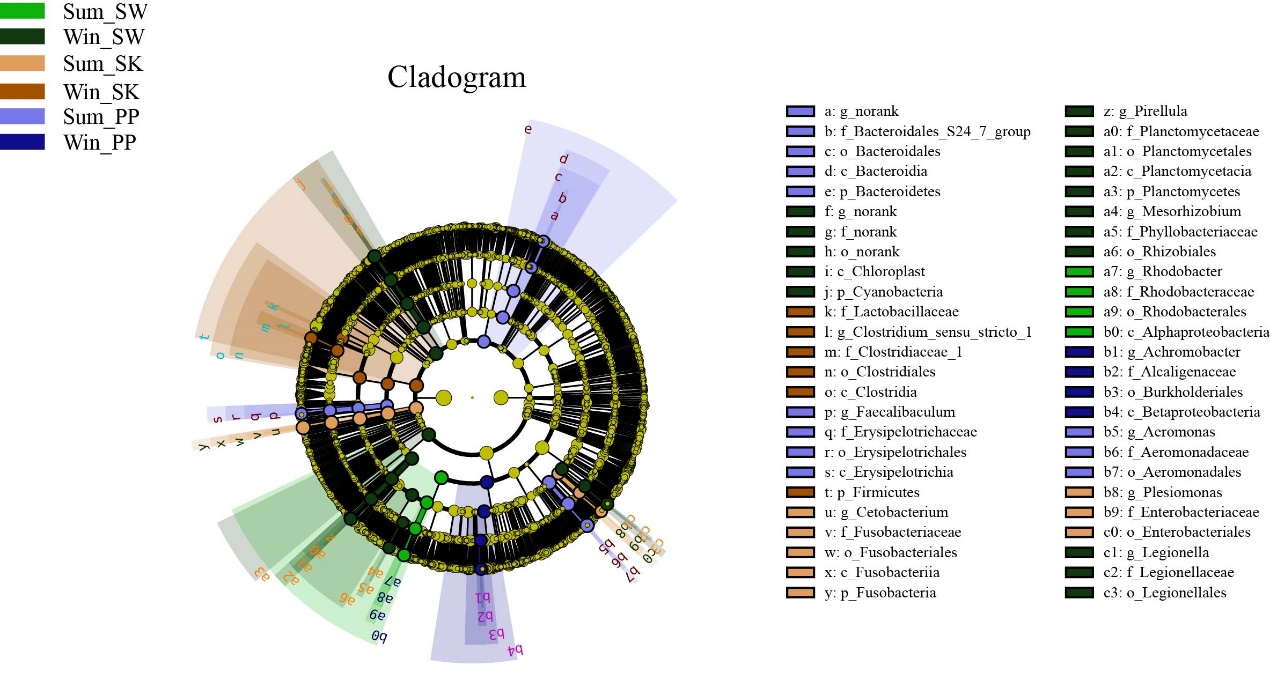


**Figure S2. Disparities of gut microbiota composition of cold-water fish between summer and winter (LDA > 4.5).**


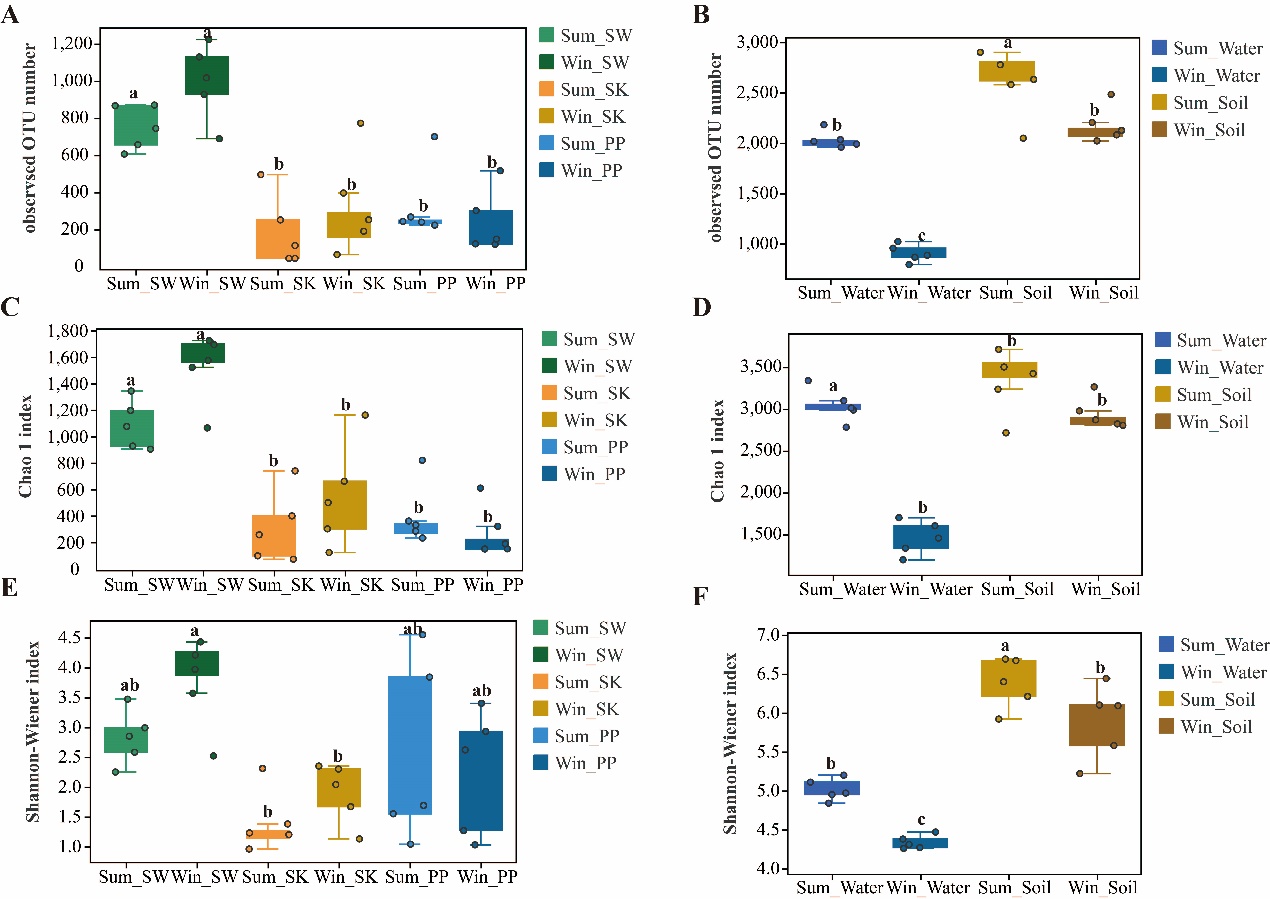


**Figure S3. Alpha diversity of gut bacteria of cold-water fish between summer and winter.**


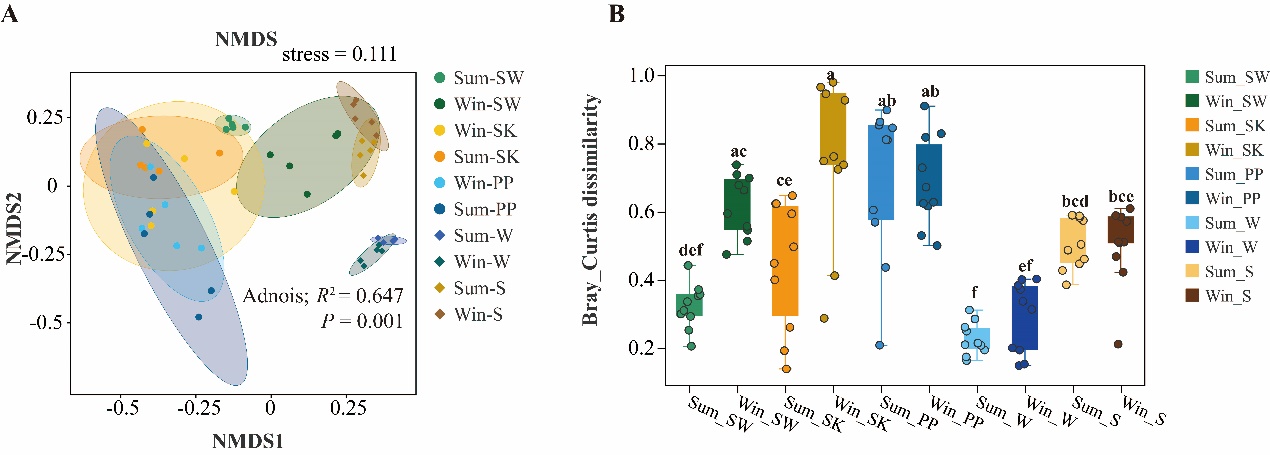


**Figure S4. Beta diversity of gut bacteria of cold-water fish and environmental bacteria between summer and winter.**


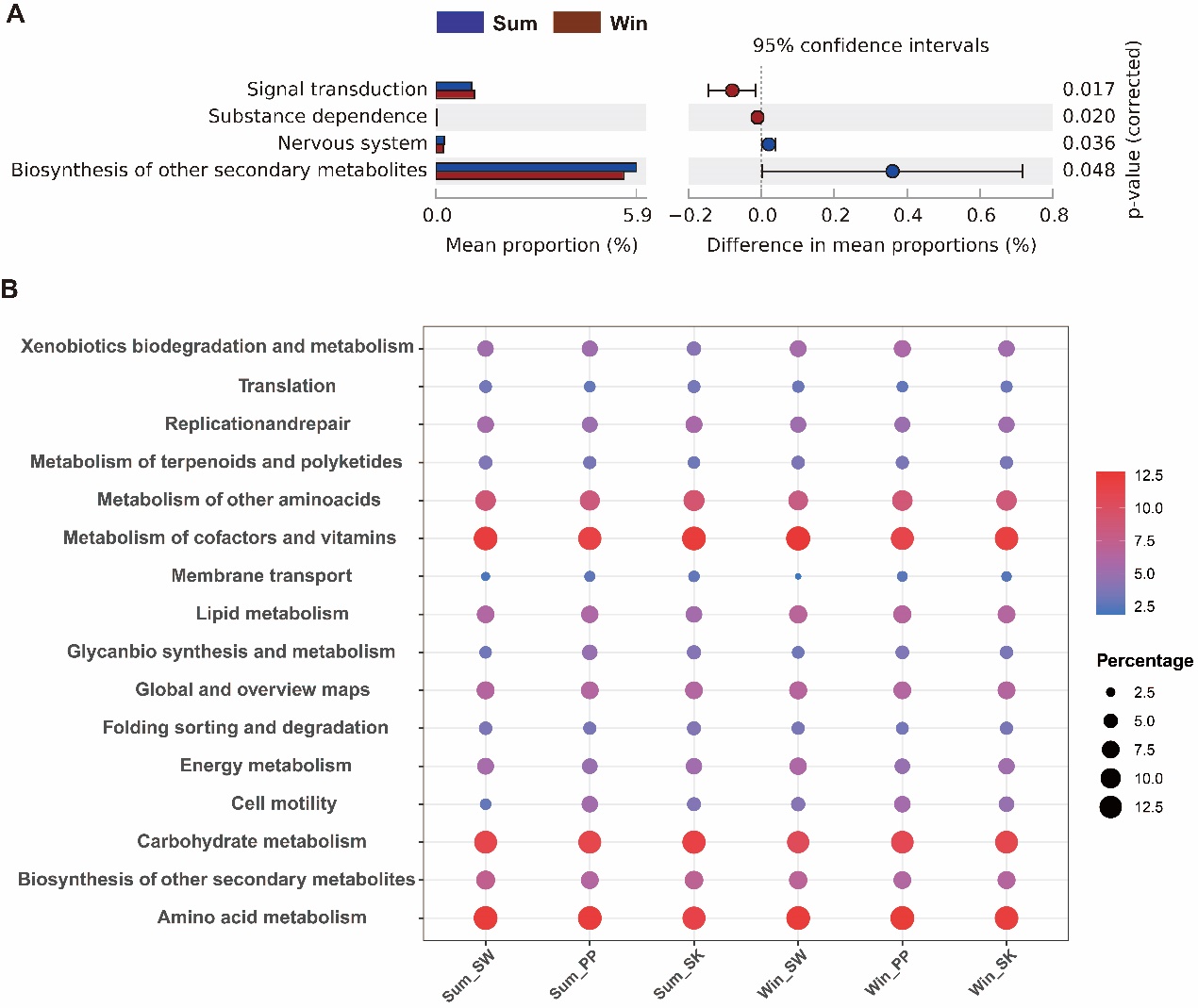


**Figure S5. Metabolic functional profiles of gut bacteria of cold-water fish between summer and winter.**


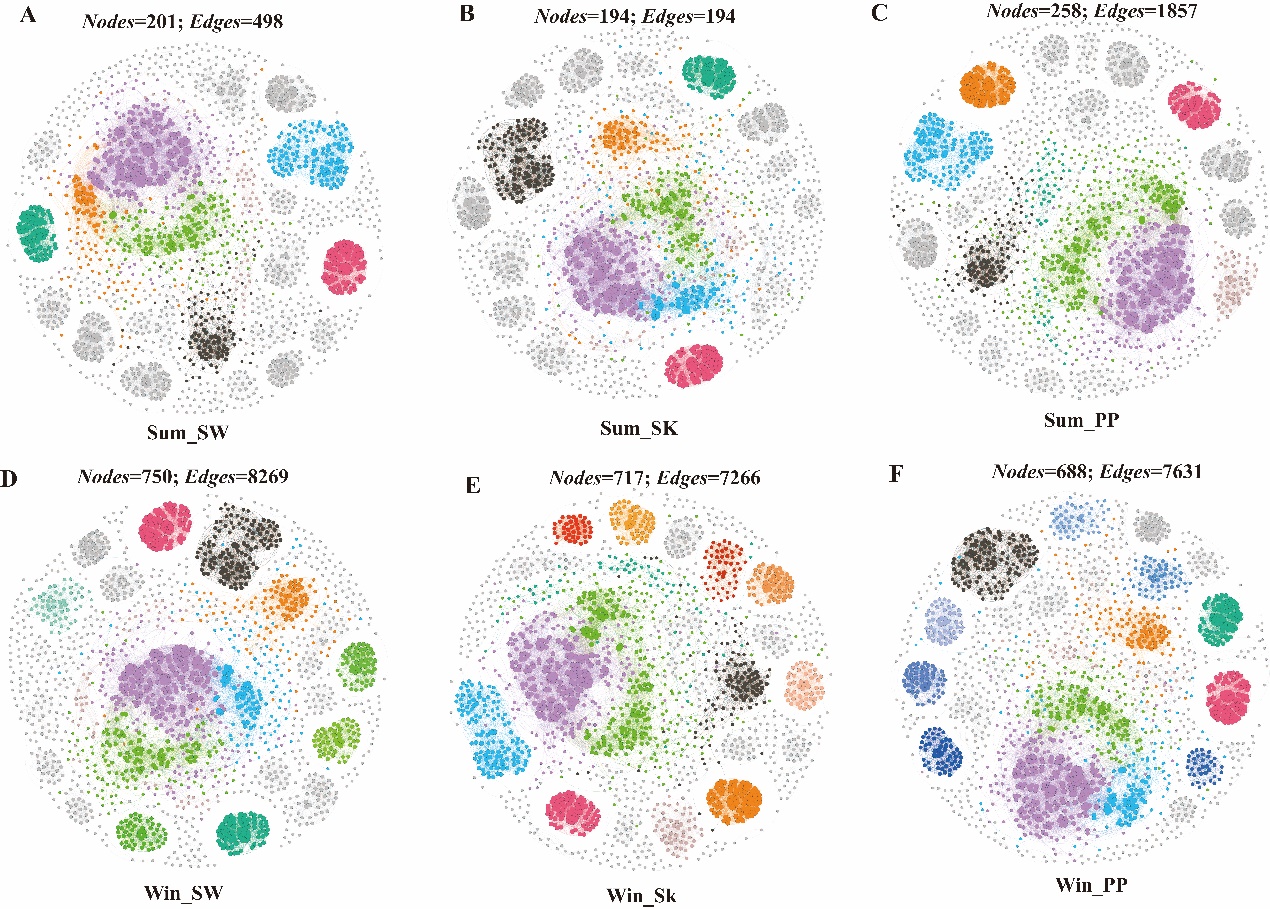


**Figure S6. Co-occurrence networks of the gut bacteria of three cold-water fishes between summer and winter.**


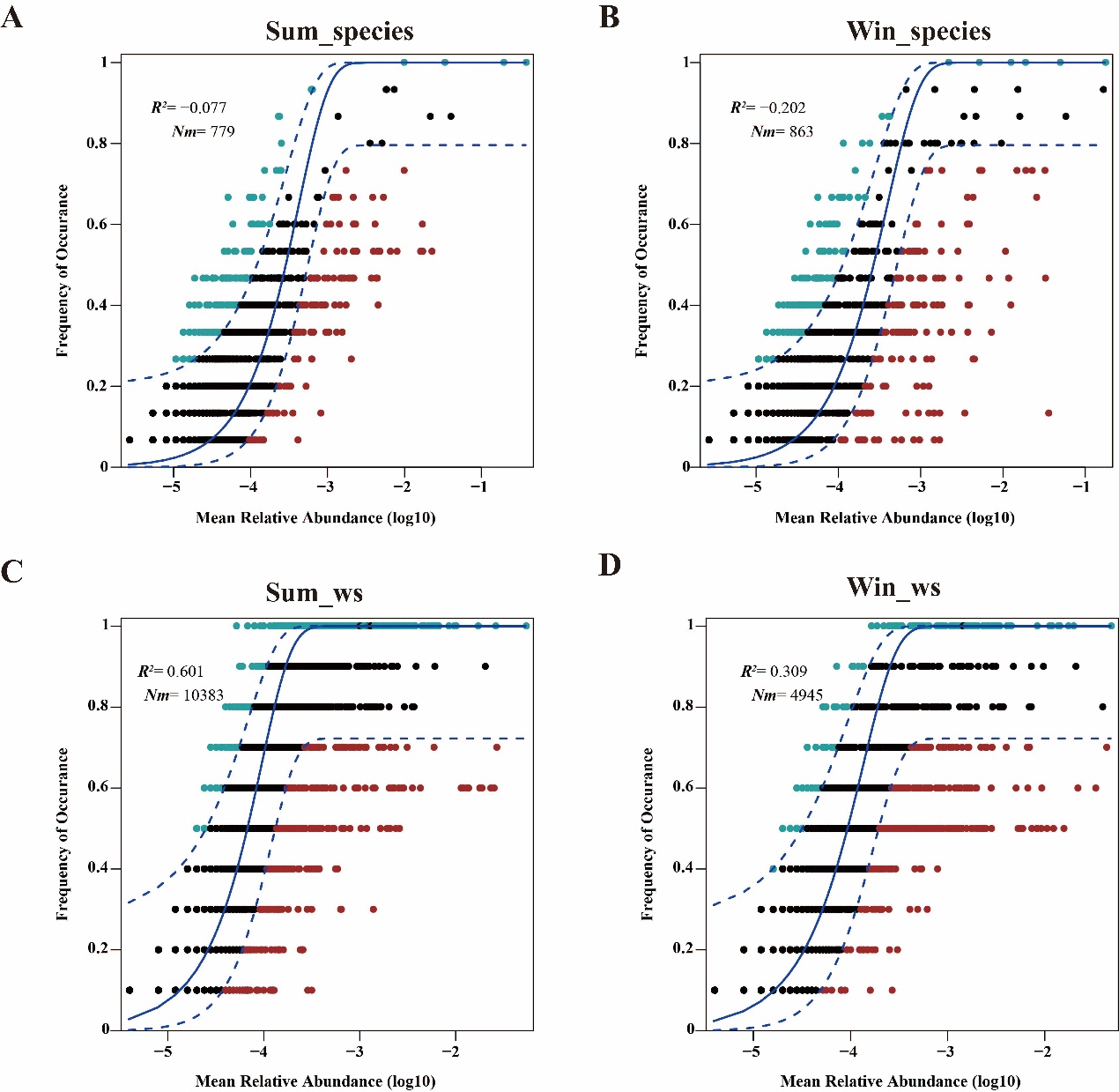


**Figure S7. Neutral community model (NCM) of the gut bacteria of cold-water fish between summer and winter.**


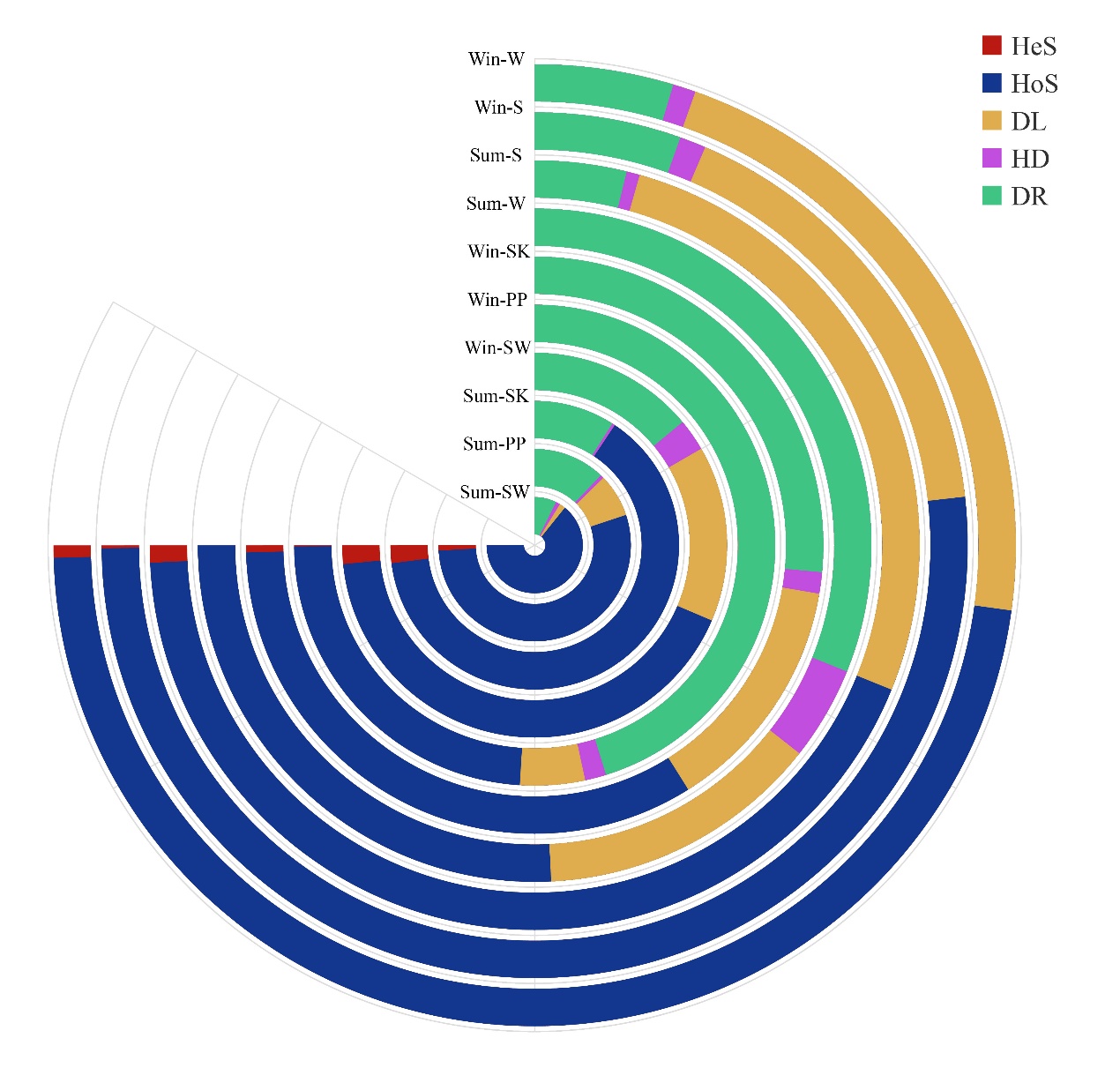


**Figure S8. Microbial assembly mechanism of the gut bacteria of three cold-water fishes and environmental bacteria between summer and winter.**


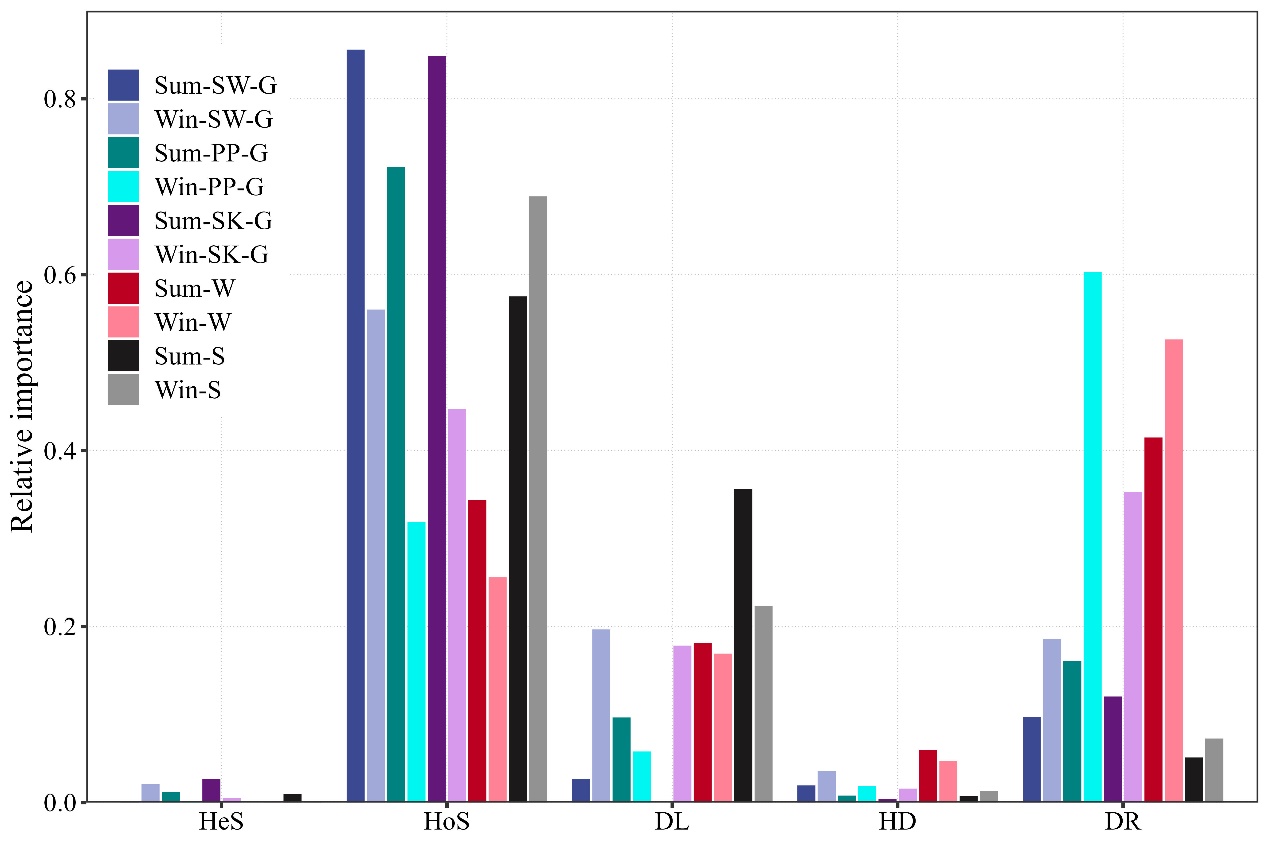


**Figure S9. Bar plots exhibiting the microbial assembly mechanism of the gut bacteria of three cold-water fishes and environmental bacteria between summer and winter.**

**Table S1. Gut bacterial composition of three cold-water fishes between summer and winter at phylum level.**

| **Phylum** | **SW** | | **SK** | | **PP** | |
| --- | --- | --- | --- | --- | --- | --- |
|  | **Sum** | **Win** | **Sum** | **Win** | **Sum** | **Win** |
| Proteobacteria | 0.267 | 0.385 | 0.300 | 0.343 | 0.559 | 0.553 |
| Fusobacteria | 0.488 | 0.043 | 0.492 | 0.262 | 0.147 | 0.206 |
| Firmicutes | 0.012 | 0.094 | 0.102 | 0.304 | 0.166 | 0.176 |
| Actinobacteria | 0.013 | 0.014 | 0.003 | 0.008 | 0.011 | 0.010 |
| Cyanobacteria | 0.119 | 0.242 | 0.092 | 0.067 | 0.003 | 0.009 |
| Bacteroidetes | 0.003 | 0.031 | 0.001 | 0.002 | 0.105 | 0.027 |
| Planctomycetes | 0.087 | 0.168 | 0.009 | 0.008 | 0.002 | 0.005 |
| Acidobacteria | 0.000 | 0.001 | 0.000 | 0.000 | 0.001 | 0.001 |
| Chloroflexi | 0.009 | 0.001 | 0.000 | 0.001 | 0.002 | 0.001 |
| Verrucomicrobia | 0.000 | 0.017 | 0.000 | 0.000 | 0.000 | 0.001 |
| Others | 0.002 | 0.005 | 0.001 | 0.005 | 0.005 | 0.011 |

**Table S2. Gut bacterial composition of three cold-water fishes between summer and winter at family level.**

| **Famliy** | **SW** | | **SK** | | **PP** | |
| --- | --- | --- | --- | --- | --- | --- |
|  | **Sum** | **Win** | **Sum** | **Win** | **Sum** | **Win** |
| Fusobacteriaceae | 0.486 | 0.043 | 0.240 | 0.491 | 0.206 | 0.147 |
| Aeromonadaceae | 0.005 | 0.002 | 0.216 | 0.207 | 0.300 | 0.365 |
| Chloroplast_norank | 0.113 | 0.233 | 0.065 | 0.092 | 0.005 | 0.002 |
| Planctomycetaceae | 0.087 | 0.167 | 0.008 | 0.009 | 0.005 | 0.002 |
| Rhodobacteraceae | 0.144 | 0.068 | 0.001 | 0.009 | 0.000 | 0.014 |
| Lactobacillaceae | 0.000 | 0.033 | 0.106 | 0.000 | 0.051 | 0.033 |
| Clostridiaceae 1 | 0.002 | 0.017 | 0.113 | 0.023 | 0.053 | 0.007 |
| Enterobacteriaceae | 0.002 | 0.004 | 0.017 | 0.056 | 0.006 | 0.049 |
| Erysipelotrichaceae | 0.002 | 0.015 | 0.007 | 0.011 | 0.013 | 0.085 |
| Carnobacteriaceae | 0.000 | 0.002 | 0.053 | 0.065 | 0.002 | 0.002 |
| Legionellaceae | 0.001 | 0.115 | 0.000 | 0.000 | 0.000 | 0.000 |
| Comamonadaceae | 0.005 | 0.022 | 0.002 | 0.001 | 0.045 | 0.021 |
| Alcaligenaceae | 0.003 | 0.004 | 0.001 | 0.000 | 0.053 | 0.027 |
| Bacteroidales S24-7 group | 0.000 | 0.011 | 0.000 | 0.000 | 0.005 | 0.057 |
| Sphingomonadaceae | 0.022 | 0.031 | 0.000 | 0.001 | 0.013 | 0.004 |
| Others | 0.128 | 0.233 | 0.171 | 0.034 | 0.242 | 0.185 |

**Table S3. Gut bacterial composition of three cold-water fishes between summer and winter at genus level.**

| **Genus** | **SW** | | **SK** | | **PP** | |
| --- | --- | --- | --- | --- | --- | --- |
|  | **Sum** | **Win** | **Sum** | **Win** | **Sum** | **Win** |
| *Cetobacterium* | 0.486 | 0.043 | 0.240 | 0.491 | 0.206 | 0.147 |
| *Aeromonas* | 0.005 | 0.002 | 0.216 | 0.207 | 0.300 | 0.365 |
| *Chloroplast_norank* | 0.113 | 0.233 | 0.065 | 0.092 | 0.005 | 0.002 |
| *Clostridium sensu stricto 1* | 0.001 | 0.014 | 0.108 | 0.023 | 0.053 | 0.007 |
| *Rhodobacter* | 0.126 | 0.063 | 0.000 | 0.007 | 0.000 | 0.001 |
| *Pirellula* | 0.027 | 0.113 | 0.001 | 0.004 | 0.000 | 0.000 |
| *Carnobacterium* | 0.000 | 0.002 | 0.053 | 0.065 | 0.000 | 0.002 |
| *Lactobacillus* | 0.000 | 0.033 | 0.000 | 0.000 | 0.051 | 0.033 |
| *Legionella* | 0.001 | 0.115 | 0.000 | 0.000 | 0.000 | 0.000 |
| *Plesiomonas* | 0.001 | 0.003 | 0.008 | 0.056 | 0.005 | 0.042 |
| *Pediococcus* | 0.000 | 0.000 | 0.105 | 0.000 | 0.000 | 0.000 |
| *Achromobacter* | 0.003 | 0.001 | 0.001 | 0.000 | 0.053 | 0.026 |
| *Bacteroidales S24-7 group_norank* | 0.000 | 0.011 | 0.000 | 0.000 | 0.005 | 0.057 |
| *Brevundimonas* | 0.001 | 0.001 | 0.001 | 0.000 | 0.046 | 0.018 |
| *Delftia* | 0.002 | 0.001 | 0.001 | 0.000 | 0.042 | 0.020 |
| *Erysipelotrichaceae_uncultured* | 0.001 | 0.007 | 0.005 | 0.010 | 0.012 | 0.024 |
| *Faecalibaculum* | 0.000 | 0.005 | 0.000 | 0.000 | 0.000 | 0.050 |
| *Pandoraea* | 0.001 | 0.001 | 0.001 | 0.000 | 0.036 | 0.016 |
| *Deefgea* | 0.000 | 0.010 | 0.033 | 0.002 | 0.001 | 0.001 |
| *Shewanella* | 0.000 | 0.000 | 0.013 | 0.012 | 0.015 | 0.003 |
| *Others* | 0.231 | 0.342 | 0.147 | 0.028 | 0.170 | 0.185 |

**Table S4. Gut bacterial alpha diversity of three cold-water fishes and environmental bacteria between summer and winter at phylum level.**

| **Species** | **Season** | **Alpha diversity** | | |
| --- | --- | --- | --- | --- |
|  |  | **observed OTUs** | **Chao 1 index** | **Shannon-Wiener index** |
| SW | **Sum** | 748 ± 120 | 1089±185 | 3±0.46 |
|  | **Win** | 997 ± 205 | 1515±266 | 4±0.75 |
| SK | **Sum** | 189 ± 191 | 312±272 | 1±0.52 |
|  | **Win** | 335 ± 272 | 549±398 | 2±0.51 |
| PP | **Sum** | 334 ± 205 | 405±237 | 3±1.6 |
|  | **Win** | 997 ± 205 | 283±195 | 2±1.05 |
| Water | **Sum** | 2035 ± 86 | 3044±201 | 5±0.14 |
|  | **Win** | 904 ± 87 | 1459±202 | 4±0.09 |
| Sediment | **Sum** | 2586 ± 327 | 3318±378 | 6±0.32 |
|  | Win | 2182 ± 181 | 2948±189 | 6±0.48 |

**Table S5. Environmental variables of gut samples of three cold-water fishes between summer and winter.**

| **Sample ID** | **pH** | **TEM** | **DO** | **US** | **TDS** | **SALT** | **ALT** |
| --- | --- | --- | --- | --- | --- | --- | --- |
| Sum-PP1 | 8.32 | 18.2 | 8.7 | 221 | 109 | 0.11 | 2009 |
| Sum-PP2 | 8.32 | 18.2 | 8.7 | 221 | 109 | 0.11 | 2009 |
| Sum-PP3 | 8.32 | 18.2 | 8.7 | 221 | 109 | 0.11 | 2009 |
| Sum-PP4 | 8.32 | 18.2 | 8.7 | 221 | 109 | 0.11 | 2009 |
| Sum-PP5 | 8.32 | 18.2 | 8.7 | 221 | 109 | 0.11 | 2009 |
| Sum-S1 | 8.43 | 11.8 | 10 | 310 | 155 | 0.15 | 1573 |
| Sum-S2 | 8.43 | 12.8 | 10 | 299 | 154 | 0.15 | 1534 |
| Sum-S3 | 8.52 | 16.2 | 10.6 | 250 | 123 | 0.12 | 1389 |
| Sum-S4 | 8.24 | 16.7 | 9.5 | 248 | 123 | 0.12 | 1337 |
| Sum-S5 | 8.24 | 16.7 | 9.5 | 248 | 123 | 0.12 | 1337 |
| Sum-SK1 | 8.32 | 18.2 | 8.7 | 221 | 109 | 0.11 | 2009 |
| Sum-SK2 | 8.32 | 18.2 | 8.7 | 221 | 109 | 0.11 | 2009 |
| Sum-SK3 | 8.32 | 18.2 | 8.7 | 221 | 109 | 0.11 | 2009 |
| Sum-SK4 | 8.32 | 18.2 | 8.7 | 221 | 109 | 0.11 | 2009 |
| Sum-SK5 | 8.32 | 18.2 | 8.7 | 221 | 109 | 0.11 | 2009 |
| Sum-SW1 | 8.43 | 11.8 | 10 | 310 | 155 | 0.15 | 1573 |
| Sum-SW2 | 8.43 | 12.8 | 10 | 299 | 154 | 0.15 | 1534 |
| Sum-SW3 | 8.49 | 15.3 | 10 | 258 | 130 | 0.13 | 1462 |
| Sum-SW4 | 8.52 | 16.2 | 10.6 | 250 | 123 | 0.12 | 1389 |
| Sum-SW5 | 8.24 | 16.7 | 9.5 | 248 | 123 | 0.12 | 1337 |
| Sum-W1 | 8.43 | 11.8 | 10 | 310 | 155 | 0.15 | 1573 |
| Sum-W2 | 8.43 | 11.8 | 10 | 310 | 155 | 0.15 | 1573 |
| Sum-W3 | 8.43 | 11.8 | 10 | 310 | 155 | 0.15 | 1573 |
| Sum-W4 | 8.43 | 11.8 | 10 | 310 | 155 | 0.15 | 1573 |
| Sum-W5 | 8.24 | 16.7 | 9.5 | 248 | 123 | 0.12 | 1337 |
| Win-PP1 | 8.88 | 4.8 | 8 | 236 | 118 | 0.11 | 2009 |
| Win-PP2 | 8.88 | 4.8 | 8 | 236 | 118 | 0.11 | 2009 |
| Win-PP3 | 8.88 | 4.8 | 8 | 236 | 118 | 0.11 | 2009 |
| Win-PP4 | 8.88 | 4.8 | 8 | 236 | 118 | 0.11 | 2009 |
| Win-PP5 | 8.88 | 4.8 | 8 | 236 | 118 | 0.11 | 2009 |
| Win-S1 | 8.87 | 9.1 | 8.9 | 291 | 147 | 0.12 | 1573 |
| Win-S2 | 8.87 | 9.1 | 8.9 | 291 | 147 | 0.12 | 1573 |
| Win-S3 | 8.87 | 9.1 | 8.9 | 291 | 147 | 0.12 | 1573 |
| Win-S4 | 8.87 | 9.1 | 8.9 | 291 | 147 | 0.12 | 1573 |
| Win-S5 | 8.85 | 8.9 | 8.7 | 233 | 118 | 0.11 | 1337 |
| Win-SK1 | 8.88 | 4.8 | 8 | 236 | 118 | 0.11 | 2009 |
| Win-SK2 | 8.88 | 4.8 | 8 | 236 | 118 | 0.11 | 2009 |
| Win-SK3 | 8.88 | 4.8 | 8 | 236 | 118 | 0.11 | 2009 |
| Win-SK4 | 8.88 | 4.8 | 8 | 236 | 118 | 0.11 | 2009 |
| Win-SK5 | 8.88 | 4.8 | 8 | 236 | 118 | 0.11 | 2009 |
| Win-SW1 | 8.87 | 9.1 | 8.9 | 291 | 147 | 0.12 | 1573 |
| Win-SW2 | 7.79 | 9 | 9 | 288 | 143 | 0.12 | 1534 |
| Win-SW3 | 8.96 | 10.5 | 8.9 | 233 | 115 | 0.11 | 1462 |
| Win-SW4 | 8.91 | 9.2 | 8.9 | 245 | 118 | 0.11 | 1389 |
| Win-SW5 | 8.85 | 8.9 | 8.7 | 233 | 118 | 0.11 | 1337 |
| Win-W1 | 8.87 | 9.1 | 8.9 | 291 | 147 | 0.12 | 1573 |
| Win-W2 | 8.87 | 9.1 | 8.9 | 291 | 147 | 0.12 | 1573 |
| Win-W3 | 8.87 | 9.1 | 8.9 | 291 | 147 | 0.12 | 1573 |
| Win-W4 | 8.87 | 9.1 | 8.9 | 291 | 147 | 0.12 | 1573 |
| Win-W5 | 8.85 | 8.9 | 8.7 | 233 | 118 | 0.11 | 1337 |

Note: “ALT” means “altitude”.
